# MP-Net: Deep learning-based segmentation for fluorescence microscopy images of microplastics isolated from clams

**DOI:** 10.1371/journal.pone.0269449

**Published:** 2022-06-15

**Authors:** Ho-min Park, Sanghyeon Park, Maria Krishna de Guzman, Ji Yeon Baek, Tanja Cirkovic Velickovic, Arnout Van Messem, Wesley De Neve

**Affiliations:** 1 Center for Biosystems and Biotech Data Science, Ghent University Global Campus, Incheon, South Korea; 2 Center for Food Chemistry and Technology, Ghent University Global Campus, Incheon, South Korea; 3 Department of Food Technology, Safety and Health, Ghent University, Ghent, Belgium; 4 Department of Chemistry, University of Belgrade, Serbia; 5 Serbian Academy of Sciences and Arts, Belgrade, Serbia; 6 IDLab, Department of Electronics and Information Systems, Ghent University, Ghent, Belgium; 7 Department of Applied Mathematics, Computer Science and Statistics, Ghent University, Ghent, Belgium; University Tunku Abdul Rahman, MALAYSIA

## Abstract

Environmental monitoring of microplastics (MP) contamination has become an area of great research interest, given potential hazards associated with human ingestion of MP. In this context, determination of MP concentration is essential. However, cheap, rapid, and accurate quantification of MP remains a challenge to this date. This study proposes a deep learning-based image segmentation method that properly distinguishes fluorescent MP from other elements in a given microscopy image. A total of nine different deep learning models, six of which are based on U-Net, were investigated. These models were trained using at least 20,000 patches sampled from 99 fluorescence microscopy images of MP and their corresponding binary masks. MP-Net, which is derived from U-Net, was found to be the best performing model, exhibiting the highest mean *F*_1_-score (0.736) and mean IoU value (0.617). Test-time augmentation (using brightness, contrast, and HSV) was applied to MP-Net for robust learning. However, compared to the results obtained without augmentation, no clear improvement in predictive performance could be observed. Recovery assessment for both spiked and real images showed that, compared to already existing tools for MP quantification, the MP quantities predicted by MP-Net are those closest to the ground truth. This observation suggests that MP-Net allows creating masks that more accurately reflect the quantitative presence of fluorescent MP in microscopy images. Finally, MAP (Microplastics Annotation Package) is introduced, an integrated software environment for automated MP quantification, offering support for MP-Net, already existing MP analysis tools like MP-VAT, manual annotation, and model fine-tuning.

## 1 Introduction

Since the 1940s, the production of plastics has increased rapidly, given the attractive properties of plastic goods (durable, lightweight, corrosion resistant) and the inexpensive methods for manufacturing them. However, this once desirable material has currently become a significant environmental concern [1]. Indeed, plastic pollution has become so severe that even an island of plastic, called the Great Pacific Garbage Patch, can now be found in the Central Pacific Gyre [2]. Furthermore, in the marine environment, microplastics (MP; < 5 mm) are the most dominant form of aquatic plastic litter [3]. They are derived from synthetic polymers that are primarily manufactured in small sizes (primary source) or from the degradation of large plastic fragments (secondary source) [4].

Given their small size, marine organisms ingest MP with their natural food during feeding. This raises several concerns regarding the safety of seafood consumption [5]. As such, MP ingestion by marine biota, in particular those intended for human consumption, is attracting more and more research attention.

Investigating MP consumption by marine biota is usually done by first extracting MP, followed by MP collection through filtration. Using a microscope, MP pieces are then manually sorted and counted. The latter steps are typically performed by multiple researchers in order to avoid measurement bias. Since this approach is a labor-intensive and time-consuming task, there is a great interest in the development of automated techniques. In this respect, a number of tools that enable semi- or fully automated quantification of MP in images are already available, such as MP-VAT [6] and MP-VAT 2.0 [7]. However, these tools suffer from drawbacks like overestimation and mislabeling, as demonstrated by the experiments presented in this paper. Therefore, a strong need exists for the development of computational tools that facilitate accurate recognition of MP in images, simultaneously measuring quantity and characterizing attributes such as size and shape.

In this paper, we propose a novel approach that leverages deep learning for mimicking the way MP is investigated by researchers, making it possible to automatically determine the quantity, size, and shape of stained MP in fluorescence microscopy images. To implement this approach, we explored several image segmentation models that make use of multi-layered artificial neural networks. To that end, we created training examples out of microscopy images, containing MP that were isolated from clams and that were subsequently stained with a fluorescent dye called Nile Red.

Specifically, in this paper, we introduce MP-Net, an effective U-Net-based deep learning model for MP segmentation. This model is part of a new software package that we have named Microplastics Annotation Package (MAP), coming with a graphical user interface (GUI) that enables user-friendly MP analyses. Furthermore, this software package embeds already existing non-deep learning tools for MP analysis (MP-VAT, MP-VAT 2.0, C-VAT, and Galaxy Count), thus also making it possible for users to select—after a comparison—the tool that best fits their research purposes.

The initial research efforts dedicated to the creation of MP-Net and MAP were first presented in a workshop paper by Baek et al. [8]. This journal paper builds on top of that preliminary work by providing a more in-depth analysis at different levels:

- More candidate models: In the previous study, we investigated three U-Net-based models. In this study, we incorporate other types of segmentation models and test-time augmentation (TTA), resulting in the fine-tuning and evaluation of a total of nine different deep learning models.
- Improved annotation: In the previous study, we made use of a dataset, further referred to as Dataset A, that was composed of 99 images. These images were labeled by a single researcher using an RGB-based thresholding method in ImageJ [9]. In this study, we make use of Dataset B, created by performing a pixel-based comparison of the labels provided by three different researchers.
- Recovery assessment: Next to a number of performance metrics commonly used in the field of computer vision, we also make use of percentage recovery, using both artificial and real-world MP, an approach that is more meaningful from the point-of-view of environmental monitoring.

## 2 Related work

The use of deep learning for the detection of MP in environmental samples has been studied before, taking as input different types of spectral data. For instance, the presence of MP in mussels was detected and characterized using a random forest classification (RFC) algorithm, using micro-FTIR and micro-Raman spectral data [10–12]. In soil, MP contamination was detected using a convolutional neural network (CNN) model that works with ultra violet—near infrared spectra (UV-NIR) [13]. However, the collection of spectral data is time-consuming and expensive, mostly due to the high cost of specialized equipment and the need for a trained operator [14]. Because of these constraints, Nile red staining became an attractive alternative for MP visualization and detection.

The aforementioned staining technique relies on the affinity of Nile red to different plastic polymers [15]. Indeed, thanks to the fluorescent nature of the dye, only a fluorescence microscope is needed to observe the MP, after which images can be captured with a camera, and where these images can then be analyzed to quantify and characterize the MP present in the sample at hand. Taken together, Nile red staining is relatively easier and cheaper to do than the collection of spectral data.

To date, several image-based techniques are available for working with unstained and Nile red-stained MP, as shown in Table 1. However, a deep learning-based method that takes advantage of Nile red-stained MP has not been investigated yet. The following section describes already existing image-based methods for MP analysis, outlining their most important features.

**Table 1:** Literature reports that describe image-based methods for MP analysis. Highlighted (bold) papers made applications publicly available to other researchers. NOAA protocol stands for National Oceanic and Atmospheric Administration protocol [16].

Authors	Tested MP size	Extraction protocol	Source	Analysis approach
Mukhanov et al. [17]	0.3 ∼ 5 mm	NOAA collect, clean, scan	Sea surface	Photoshop (TR) ImageJ (MC, SC, SM)
Lorenzo-Navarro et al. [18]	1 ∼ 5 mm	NOAA collect, clean, scan	Beach sediments	Adaptive thresholding (TR) Machine learning (SC)
Lorenzo-Navarro et al. [19]	1 ∼ 5 mm	NOAA collect, clean, photograph	Beach sediments	U-Net segmentation (TR) VGG-16 (SC)
Massarelli et al. [20]	0.5 ∼ 5 mm	NOAA collect, clean, photograph	River water, sediments	OpenCV (MC, SM) Machine learning (SC)
Maes et al. [21]	2 ∼ 20 μm	Nile Red	Beach sediments	ImageJ (Polymer classification)
**Mason et al**. [22]	6.5+ μm	Nile red	Bottled water	Galaxy Count (TR, MC, SM)
**Prata et al**. [6]	0.65+ μm	Nile red	River water, sediments	ImageJ (MC, SC, SM) Max entropy (TR)
**Prata et al**. [7]	2+ μm	Nile red	River and lagoon water, sediments	ImageJ (MC, SC, SM) Renyi entropy (TR)
Patchaiyappan et al. [23]	100 ∼ 1, 000 μm	Nile red	Beach sediments	ImageJ (MC, SC, SM)
Dowarah et al. [24]	21 ∼ 1, 500 μm	Nile red	Bivalves	ImageJ (MC, SC, SM)
**Chen et al**. [25]	7.0+ μm	Nile red	Beverage containers	Absolute thresholding (TR) ImageJ (MC, SC, SM)
**Our approach**	10 ∼ 1, 000 μm	Nile red	Bivalves	U-Net segmentation (TR) MAP (MC, SC, SM)

* TR: Thresholding, MC: MP counting, SC: Shape classification, SM: size measurement.

### 2.1 Image-based methods

The image-based methods shown in Table 1 can be mainly classified based on the type of input image used: unstained or Nile red-stained. The first four studies made use of unstained images, while the remaining seven studies utilized Nile red staining to produce images of fluorescent MP. Each method has its own features, which are summarized in the last column of Table 1. These features can be one or a combination of the following:

- thresholding (TR);
- MP counting (MC);
- shape classification (SC); and
- size measurement (SM).

**Fig 1:**
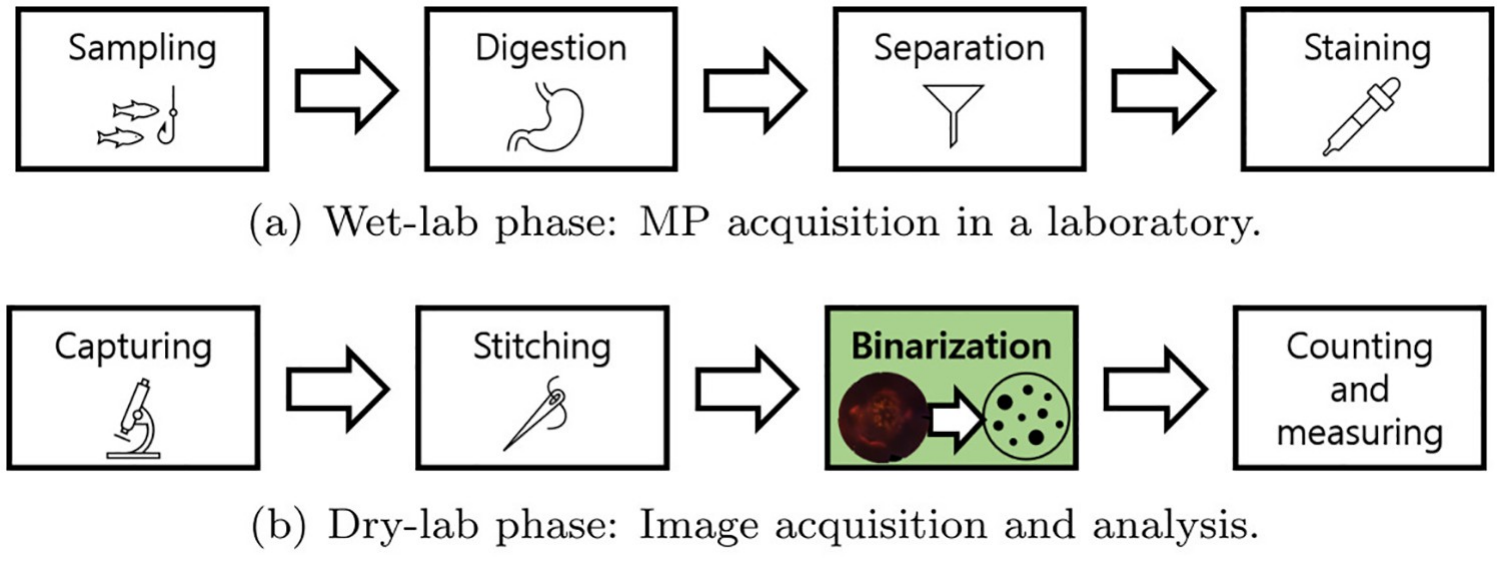
Overview of MP measurement. Overall approach towards MP measurement, from sampling to image processing: (a), (b) corresponds to the complete MP measurement process we made use of. We propose a new binarization method (green-colored box) that uses deep learning. (a) Wet-lab phase: MP acquisition in a laboratory. (b) Dry-lab phase: Image acquisition and analysis.

TR corresponds to the binarization step shown in Fig 1. This technique determines whether pixels are MP (foreground) or non-MP (background). MC determines the quantity of MP present in the mask that is the result of TR, while SC classifies the shape of each piece of MP. In general, we can make a distinction between three types of shapes, as described in Section 3.2: particle, fragment, and fiber. Finally, SM measures the size of each piece of MP. To that end, the Feret diameter (μm) or the occupying area (μm^2^) are used. The Feret diameter is defined as the length of the longest straight line that can be drawn in a given piece of MP.

In the studies presented in [17–20], which made use of unstained images, the MP can be distinguished by the naked eye (0.3 ∼ 5 mm). The National Oceanic and Atmospheric Administration (NOAA) protocol [16] was used in these studies. This protocol is generally adopted to isolate MP debris from the sea surface and sediment, and to subsequently determine size and shape. In contrast, the remaining studies that employed Nile red observed smaller MP (2 ∼ 1,500 μm). Due to the high fluorescence intensity of the dye, smaller MP, which are typically difficult to distinguish, can be noticed easily.

Among the studies that involved unstained MP, the authors of [18–20] proposed a machine learning model, trained with unstained and large pieces of MP. For stained MP images, the initial work we presented in [8] showcased the first deep learning approach. The majority of the other existing methods are implemented in ImageJ, an open-source software package for scientific image analysis. Only the work by [22] introduced a new platform, called Galaxy Count, which is a semi-automated tool requiring user input. In 2019, [6] introduced MP-VAT, the first automated MP analysis tool that works in ImageJ. In 2020, an upgraded version of MP-VAT, called MP-VAT 2.0, was released [7]. A modified version of MP-VAT was introduced in 2021 by [25], which will be further referred to as C-VAT for the sake of convenience. Unlike MP-VAT, C-VAT copes with noise by using a despeckle filter and outlier removal, also using an absolute threshold of 222 to distinguish background from MP. Being the most recent and high-throughput MP analysis tools for stained MP images, the effectiveness of MP-VAT, MP-VAT 2.0, and C-VAT is compared to the effectiveness of our deep learning models in Section 5.

A common feature of Galaxy Count, MP-VAT, MP-VAT 2.0, and C-VAT is the use of global TR, which is the main decision-making function that determines if a pixel in an image is classified as MP or non-MP. The following two sections describe in detail global TR and the differences with deep learning-based TR models.

### 2.2 Differences between TR-based models

The approaches followed by the four studies highlighted in bold in Table 1 [6, 7, 22, 25] are publicly available. Although these approaches use almost identical methods for MC, SM, and SC, considerable differences can be observed for TR, as discussed below.

In what follows, we make use of the notation M to refer to a TR-based model that is responsible for MP and background separation. M takes as input a microscopy image $I\in R^{w\times h\times c}$ and returns a mask $M\in R^{w\times h}$, with *w* denoting the image width, *h* the image height, and *c* the number of color channels:

$$\begin{array}{c} M=M(I)\;. \end{array}$$ (1)

Note that a color image usually comes with three channels (Red, Green, Blue; RGB; *c* = 3). If *c* is not mentioned explicitly, then a single-channel image (i.e., a gray-scale image; *c* = 1) is used.

The TR-based model M can be split into two sub-models. The first sub-model *f*_*I* → *G*_ takes *I* as an input and outputs a one-dimensional monotonic intensity image $G\in R^{w\times h}$. The second sub-model *f*_*G* → *M*_ takes G as an input and outputs the corresponding mask *M*. For *f*_*G* → *M*_, a thresholding function can be used, like the one shown in the equation below:

$$\begin{array}{c} M_{ij}=\{\begin{array}{cc} 255, & \text{if}\;G_{ij}>T\;\\ 0, & \text{if}\;G_{ij}\leq T\; \end{array},\;i=1,\ldots ,w;\;j=1,\ldots ,h\;, \end{array}$$ (2)

with *T* denoting the threshold value. If a pixel has a value of 255, it is assumed to denote MP, and if a pixel has a value of 0, it is assumed to denote background. This approach, which can be referred to as global thresholding or simple thresholding, allows extracting objects of interest (i.e., microplastics) from an image by making use of a single fixed threshold value *T* for all pixels that can be found in the given image of size *w* × *h*. Table 2 shows the different *f*_*I* → *G*_ and *f*_*G* → *M*_ models used by the research efforts previously highlighted in Table 1.

**Table 2:** Differences between TR-based models. All existing methods consist of a channel extracting step (*f*_*I* → *G*_) and a thresholding step (*f*_*G* → *M*_).

Authors	M	*f*_*I* → *G*_	*f*_*G* → *M*_
Mason *et al*. [22]	Galaxy Count	Gray scale	Custom threshold
Prata *et al*. [6]	MP-VAT	Gray scale	Max entropy
Prata *et al*. [7]	MP-VAT 2.0	Red channel from RGB	Renyi entropy
Chen *et al*. [25]	C-VAT	Gray scale	Absolute threshold

In the column labeled ‘*f*_*I* → *G*_’ of Table 2, the value *Gray scale* means that color images have been converted to gray scale. Unlike the other three models shown in Table 2, MP-VAT 2.0 extracts and uses only the red channel of an RGB image, given that Nile red dye contains more red information. Next, in the column labeled ‘*f*_*G* → *M*_’, the value *Custom threshold* means that a user needs to iteratively adjust the threshold per image (based on direct inspection of the generated output). Similarly, *Absolute threshold* refers to thresholding with a value that is manually determined through trial and error, and where this value is used for every image. When using *Max entropy*, a threshold is selected that leads to the largest entropy after summing. *Renyi entropy* was introduced by [26], determining a final threshold by weighing three different max entropy values, calculated by setting *α* to 0.5, 1, and 2 in the following Renyi entropy equation:

$$\begin{array}{c} H_{\alpha }\left(q_{1},q_{2},...q_{n}\right)=\frac{1}{1-\alpha }\text{log}(\sum_{k=1}^{n}q_{k}^{\alpha }). \end{array}$$ (3)

Here, *q*_*k*_ denotes the normalized occurrence of pixel value *k*. In other words, *q*_*k*_ refers to the number of pixels having a brightness of *k* divided by the total number of pixels. For the case of *α* = 1, a converging Taylor series is obtained (*α* = 1 + *ϵ*, *ϵ* → 0), and the resulting expression corresponds to the Max entropy: $H_{1}=\sum_{k=1}^{n}q_{k}log\left(q_{k}\right)$.

In both cases, a large entropy is indicative of a large amount of available information. In other words, a large entropy means that MP pixels can be distinguished well from non-MP pixels. Both max entropy and Renyi entropy can be used in ImageJ (Available at https://imagej.net/plugins/auto-threshold).

The use of TR has the advantage of being intuitive and fast. However, because only one value is used to distinguish MP pixels from background pixels, it is difficult to properly recognize ambiguous pixels. In particular, fluorescence images have the property of shining brighter when dyed objects are densely gathered.

**Fig 2:**
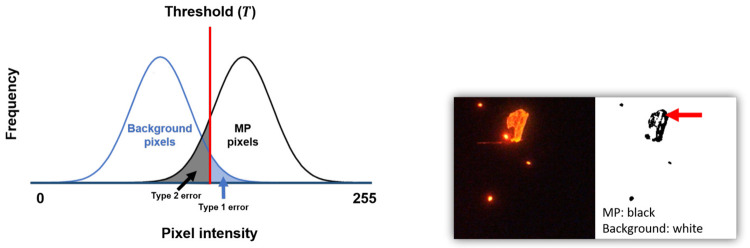
Type 1 and Type 2 errors when using a single value for thresholding.

For example, a background pixel can be wrongly recognized as an MP pixel (Type 1 error) when using a low threshold and when the pixel in question is brighter than the surrounding pixels. Alternatively, as illustrated in Fig 2(b), when the center of an MP particle is photographed relatively dark due to its thickness, a corresponding MP pixel may be recognized as a background pixel (Type 2 error). Therefore, users that apply single-value thresholding should have a good awareness of these weaknesses. Furthermore, depending on the input image used, interactive (i.e., manual) post-processing may be required for accurate MC, SC, and SM.

### 2.3 Deep learning-based TR models

Unlike already existing TR-based models that make use of global thresholding, our study focuses on building a TR-based deep learning model *f*_*I* → *M*_(*I*;*θ*) that distinguishes MP pixels from background pixels. This model creates a mask *M* directly from a given image *I*, thus not making use of distinct sub-functions (end-to-end learning).

Given an image dataset $D={\left(I_{k},M_{k}\right)}_{k=1}^{n}$, where $I_{k}\in R^{w\times h\times c}$ is an original image and where $M_{k}\in R^{w\times h}$ is its corresponding mask, let M be a segmentation model that takes *I*_*k*_ as input and predicts $\hat{M_{k}}\in R^{w\times h}$ as an approximation of the true mask *M*_*k*_:

$$\begin{array}{c} M\left(I_{k};\theta \right)={\hat{M}}_{k}\approx M_{k}\;, \end{array}$$ (4)

where *θ* are the model parameters. The difference between the predicted mask ${\hat{M}}_{k}$ and the ground truth mask *M*_*k*_ is quantified by a loss function *L*. Given a segmentation model M and a loss function *L*, we want to find the values for *θ* that minimize the total loss based on the dataset D.

Ideally, when a new image *I*_*l*_ comes in, the trained model M makes a prediction ${\hat{M}}_{l}$ that is close to the true underlying segmentation mask. Intuitively speaking, the TR-based model then mimics the MP and background selection criteria used by a researcher, given that this TR-based model has been trained on the image and mask pairs previously created by researchers.

Deep learning-based image segmentation has the disadvantage of being slow, both during training and prediction, requiring more computational power than already existing TR-based models. However, deep learning-based image segmentation has the advantage that it reduces the amount of post-processing needed (e.g., manual correction of annotations). Supported by experimental results, a more detailed comparison between already existing TR-based models and deep learning-based image segmentation models will be provided in Section 5.

## 3 Methods

This section describes the process of MP acquisition and analysis, from sampling to image processing. The entire process is composed of eight steps that are presented in Fig 1. The different steps are explained by dividing the overall process into two major phases: the wet-lab phase, as shown in Fig 1(a), and the dry-lab phase, as shown in Fig 1(b). The image pairs (fluorescent and mask) obtained at the end of the dry-lab phase are, in a next step, fed into the dataset preparation phase, as discussed in Section 4.1.

### 3.1 Wet-lab phase

#### Sampling

**Fig 3:**
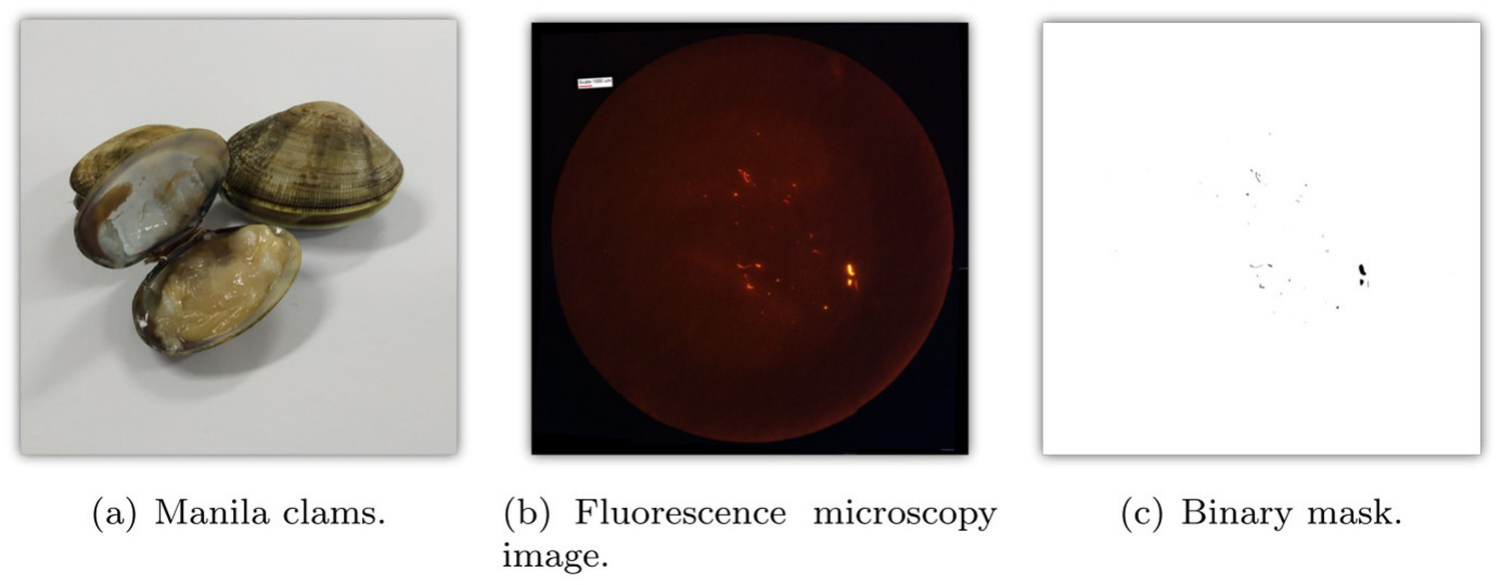
(a) Manila clams used in our experiments, (b) microscopy image of MP stained with Nile red dye, and (c) binary mask for (b). The white rectangle that can be seen in the left-upper part of (b) is used for calibration purposes. In (c), black pixels belong to MP (foreground), whereas white pixels denote non-MP (background).

Manila clam samples (*Ruditapes philippinarum*), as can be seen in Fig 3(a), were bought at the Incheon Complex Fish Market (Incheon, Korea) in May 2019. Immediately after the purchase, the samples were kept on ice. After transport to the laboratory, the clams were wrapped in aluminum foil and stored in a -20°C freezer.

#### Extraction (digestion)

All steps were performed inside a clean bench and all solutions were filtered using a 0.22μm membrane filter to avoid contamination. For sample preparation, frozen whole clam tissue was separated from the shell using a scalpel. To prevent MP from being released due to thawing, this step was executed as quick as possible. The wet weight of each clam was recorded. To dissolve organic matter, samples were incubated in 250 ml 10% KOH at 60°C with stirring for 24 hours. Through this digestion step, the MP that were inside the organism were obtained [27]. For clams weighing more than 10 g, the tissue was randomly split into two similar pieces and digested separately. Once digestion was completed, samples were vacuum filtered using GF/A (glass microfiber) filters.

#### Purification (separation)

After digestion and filtration, the MPs were separated from marine contaminants (sand, silt, and shell) through density separation. Filter papers were resuspended in 10 ml 1.37 g mL^-1^ zinc chloride with sonication for 3 mins. After three repetitions, the solutions were centrifuged and the supernatant was filtered to recover MP [21].

#### Staining

Nile Red (1 *μ*g mL^-1^ in acetone) was added to the purified sample solution at a final concentration of 10 *μ*g mL^-1^. The dye was incubated with the sample at 60°C for 30 mins with constant mixing. Solutions were vacuum filtered using a GF/A filter. To remove excess dye, the GF/A filters were washed with absolute ethanol three times. Additional washing was performed for samples that showed high background fluorescence [21].

### 3.2 Dry-lab phase

#### Capturing

Stained MP debris on filter paper was viewed under a stereomicroscope (Olympus SZX10). This stereomicroscope was equipped with a SZX2-FGFPHQ filter set having 460–480 nm excitation and 495–540 nm emission. Because each filter was too large to be captured as a single photo, images of different filter sections were taken using a DigiRetina 16 microscope camera, with the images having a resolution of 1600 × 1200 and an exposure time of 80 ms.

#### Stitching

To create a complete image of a sample, images containing different sections of the same filter paper were combined using Microsoft Image Composite Editor. That way, a total of 99 composite filter paper images were obtained from the Manila clam samples used. A representative image can be found in Fig 3(b).

#### RGB thresholding and binarization

Each composite image was loaded in ImageJ. First, the scale was set (Analyze > Set Scale) and a representative fluorescent MP piece was selected to determine the optimal RGB threshold (Image > Adjust > Color threshold) for automated MP selection. When necessary, RGB values were adjusted manually to ensure selection of all MP. Afterwards, a “mask” image was generated (Edit > Selection > Create mask), as illustrated in Fig 3(c).

#### Counting and measuring

The Microplastics Automated Counting Tool (MP-ACT) macro [6] in ImageJ was used to automatically quantify the MP in each mask (Plugins > Macros > MP-ACT). This macro utilizes ImageJ functionalities such as circularity (range goes from 0.0 to 1.0) to perform shape identification and Feret diameter (provides an approximation of the longest dimension) to perform size measurement. The shapes that can be identified include fiber (0.0–0.3), fragment (0.3–0.6), and particle (0.6–1.0). MP-ACT creates an output file that contains a tabulated summary of each MP piece that could be found in the generated mask, describing its shape and size.

## 4 Experimental design

**Fig 4:**
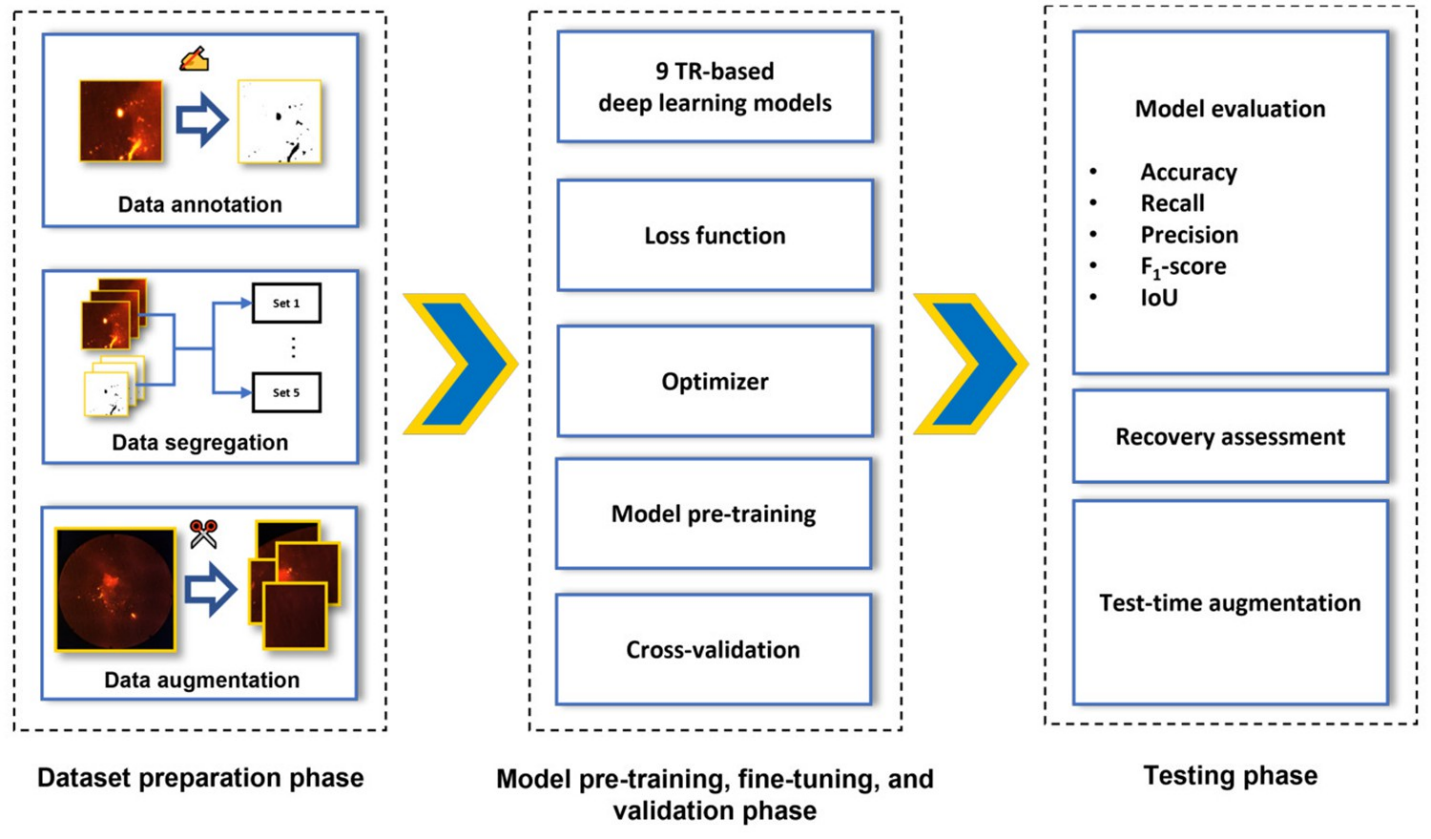
Overview of the overall approach. This approach can be divided into three phases: (1) dataset preparation, (2) model pre-training, fine-tuning, and validation, and (3) model testing.

As shown in Fig 4, the process of generating a TR-based deep learning model can be roughly summarized by making use of three major steps: (1) dataset preparation; (2) model pre-training, fine-tuning, and validation; and (3) model testing. Aside from the common performance metrics described in Section 4.3, recovery assessment, an additional measure of model effectiveness, is introduced in Section 4.4.

### 4.1 Dataset preparation

The first step, namely *Dataset preparation*, involves preparing the image and mask pair dataset D and dividing it into five subsets for the purpose of 4-fold cross-validation and testing, making sure no data leakage is occurring.

In our previous study [8], only one researcher performed labeling using RGB thresholding. For the study presented in this paper, the previously obtained thresholded RGB masks were used as a starting point. Three researchers manually and independently performed individual pixel annotation (IPA) for each image, as discussed in more detail in S1.1 Section in S1 File. The final mask for each image was subsequently obtained by making use of pixel-wise majority voting, an approach also described in more detail in S1.1 Section in S1 File. These final masks were then used as the ground truth. A total of 99 ground truth masks, corresponding to the 99 fluorescence microscopy images, were created. It should be clear that, by taking into account the input of three different researchers, labeling can be expected to be more reliable and objective. For the sake of convenience, the dataset used in our previous study [8] is denoted as Dataset A, and the dataset newly created for this study is denoted as Dataset B.

For both Dataset A and Dataset B, the 99 fluorescence images were distributed over the four cross-validation folds (further referred to as CVF (1), CVF (2), CVF (3), and CVF (4)) and the test set in such a way that the amount of MP pixels is evenly distributed. This was achieved by first arranging the ground truth masks according to an increasing number of MP pixels and by then distributing them one by one over the four cross-validation folds and the test set. In particular, we started with the test set, followed by CVF (1), CVF (2), CVF (3), and then CVF (4), after which the order was inverted. This process was repeated until all images were assigned to a particular set. That way, we prevented a particular set from containing images with a larger MP composition than the others. In the end, 19 images were assigned to the test set while each CVF contained 20 images. Note that, since the ground truth masks from Dataset A were further annotated (i.e., refined) to obtain Dataset B, the order of the images, according to an increasing number of MP pixels, was different between both datasets. As a result, the masks making up each set were also different between the two datasets, as illustrated by Table 3.

**Table 3:** Overview of the datasets used. The last two columns describe the patches sampled using a sliding window approach. Other values are expressed as mean ± standard deviation.

Dataset	Split	Number of images	Width	Height	MP (10^−2^)%	Patches created before random deletion	
						With MP	Without MP
**A**	**Test set**	19	3380.1±1523.1	2456.8±1113.1	0.010±0.013	20640	156150
	**CVF (1)**	20	3299.4±1473.5	2558.8±1282.6	0.012±0.015	29798	161496
	**CVF (2)**	20	3567.7±1704.0	2510.0±1610.0	0.015±0.017	34799	179759
	**CVF (3)**	20	2894.5±1760.2	2387.8±1670.3	0.014±0.016	34027	149959
	**CVF (4)**	20	3474.0±1638.5	2474.9±1350.9	0.012±0.018	30854	169608
**B**	**Test set**	19	2949.6±1709.7	2425.6±1594.5	1.00±1.11	30486	
	**CVF (1)**	20	3558.3±1718.0	2747.7±1630.5	1.40±1.97	29793	
	**CVF (2)**	20	3041.5±1486.7	2433.4±1209.3	1.30±1.62	30700	
	**CVF (3)**	20	3620.0±1425.1	2420.4±1247.6	1.28±1.55	33490	
	**CVF (4)**	20	3424.8±1730.0	2359.5±1362.6	1.30±1.50	26708	

**Fig 5:**
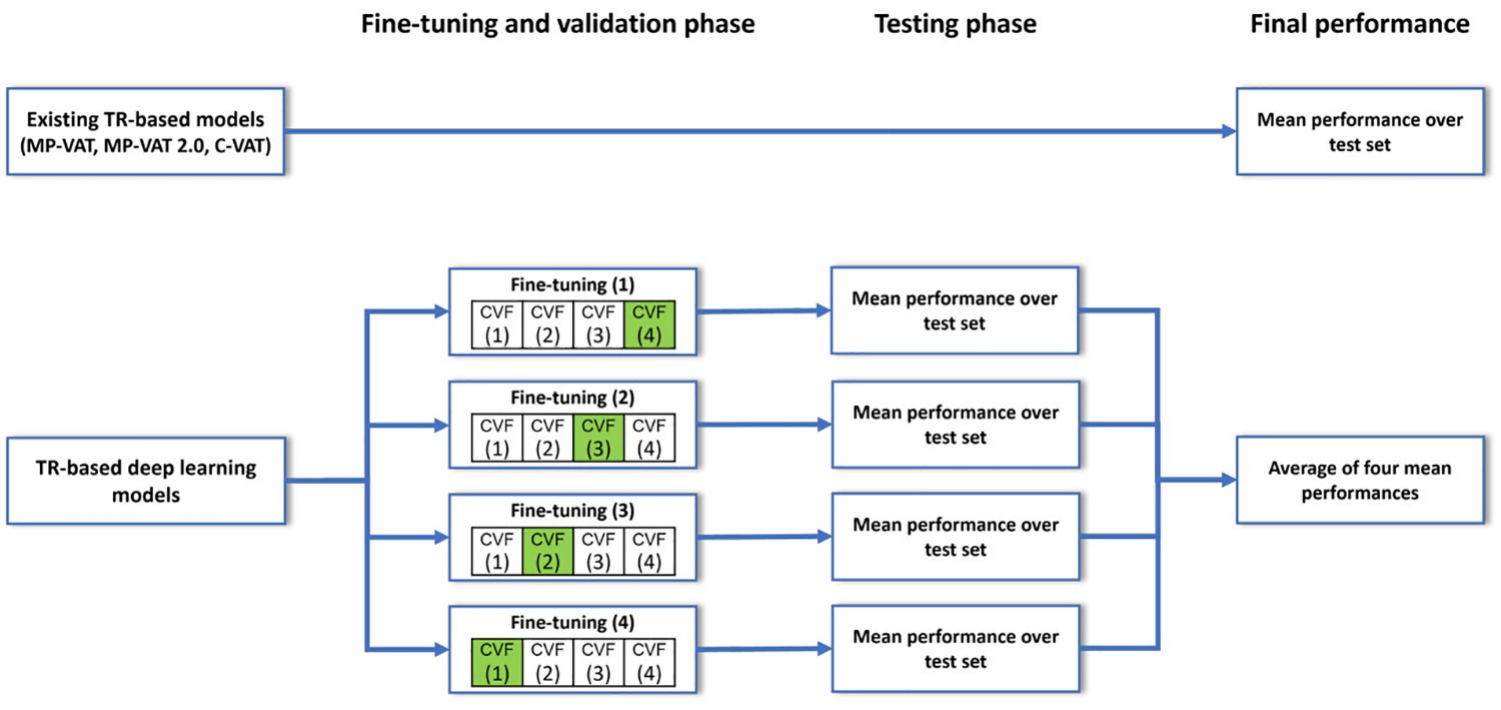
Dataset segregation. Dataset segregation for fine-tuning, validation, and testing of the already existing TR-based models and the TR-based deep learning models. Green-colored boxes refer to the fold used for validation, while white-colored boxes denote the folds used for fine-tuning.

As shown in Fig 5, CVF (1)—(4) were used for either fine-tuning or validation. For instance, for Fine-tuning (1), CVF (4) was used as the validation set (indicated as a green box), while the remaining three folds were used as the fine-tuning set (indicated as white boxes). As can be seen in the columns ‘Width’ and ‘Height’ of Table 3, our images do not have the same resolution (given the use of stitching). Since a deep learning model can only receive input of a fixed size, small patches $p\in R^{256\times 256}$ were sampled from each image *I* using a sliding window approach.

Finally, detailed information about the sampled patches created after the dataset preparation phase can also be found in Table 3, in the column labeled ‘Patches created before random deletion’. We can observe that, for Dataset A, the number of patches containing MP is significantly smaller than the number of patches without MP. This can be interpreted as our dataset having more background pixels than MP pixels. To solve this imbalance problem, we adopted over-sampling, with Dataset B not including patches without MP. A detailed description of our sliding window approach and oversampling is provided in S1.2 Section in S1 File.

### 4.2 Model pre-training, fine-tuning, and validation

**Fig 6:**
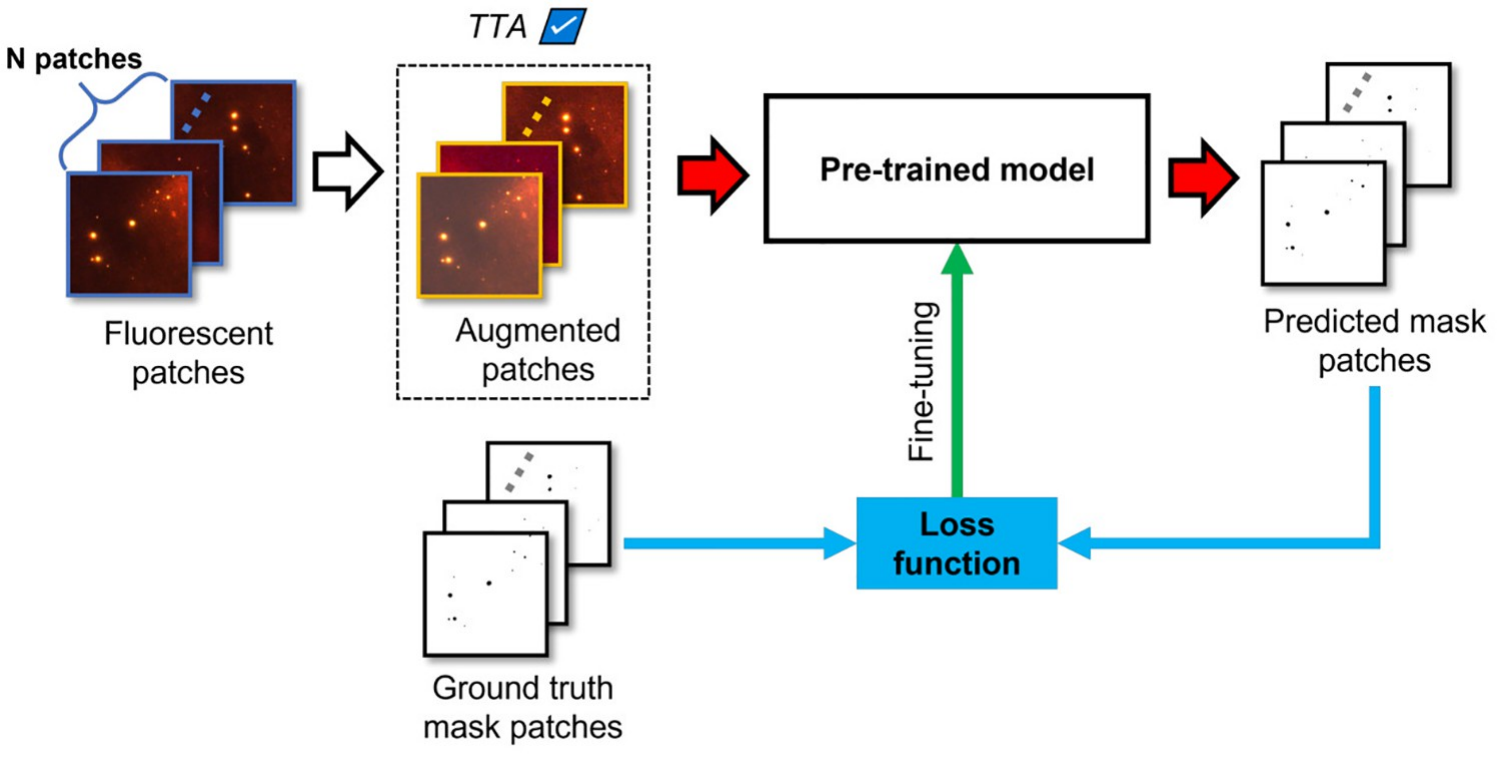
Fine-tuning of a TR-based deep learning model. *N* corresponds to the size of the mini-batch of images fed to the model. The dotted box represents an additional step that is performed when TTA is included.

This section discusses the way our TR-based deep learning models were created. As can be seen in Fig 6, and as explained in Section 2.3, images are first cropped to patches of the same size of 256 × 256. The obtained patches are then fed to a TR-based deep learning model, resulting in a predicted mask for each patch (Fig 6, red arrows). In Fig 6, *N* represents the size of the mini-batch, i.e., the number of images that is fed to the model at the same time. Next, the loss, which quantifies the difference between the ground truth mask and the predicted mask, is calculated (Fig 6, blue arrows and blue box). Based on the calculated loss, the model parameters *θ* are updated by relying on stochastic gradient descent and backpropagation [28] (Fig 6, green arrow). That way, it is possible for a TR-based deep learning model to iteratively learn how to predict masks that, after each step, are closer to the ground truth masks.

In our experiments, nine different TR-based deep learning models were evaluated by making use of Dataset B. Information about the loss and optimizer used by each model is presented in Section 5.1.2. U-Net (1) to U-Net (4) all have the same U-Net structure [29], but the loss and optimizer combinations are different. TTA U-Net (3) and TTA U-Net (4) are models that apply TTA to U-Net (3) and U-Net (4), respectively. Finally, in order to obtain a more complete performance comparison, segmentation models different from U-Net were also tested, namely FCN [30], DeepLabv3 [31], and Nested U-Net [32].

Each of the nine TR-based deep learning models consists of an encoder that extracts features from a patch and a decoder that reconstructs the patch as a mask. We initialized the encoder through transfer learning [33], making use of pre-trained parameters from two benchmark datasets, namely ImageNet [34] and MS COCO 2017 [35]. Afterwards, Dataset B was used to fine-tune the parameters of each model. Specifically, as shown in Fig 5, 4-fold cross-validation was performed. Finally, each fine-tuned model was evaluated through the respective test set and the average of the four results was taken to obtain the final effectiveness.

More details on the fine-tuning process are provided in S2.1 Section in S2 File. Information on the used models is included in S2.2 Section in S2 File, and a detailed description of the loss and the optimizer used can be found in S2.3 Section in S2 File and S2.4 Section in S2 File, respectively. Transfer learning is discussed in S2.5 Section in S2 File, while the learning curves for each model are included in S3 File.

### 4.3 Model testing

**Fig 7:**
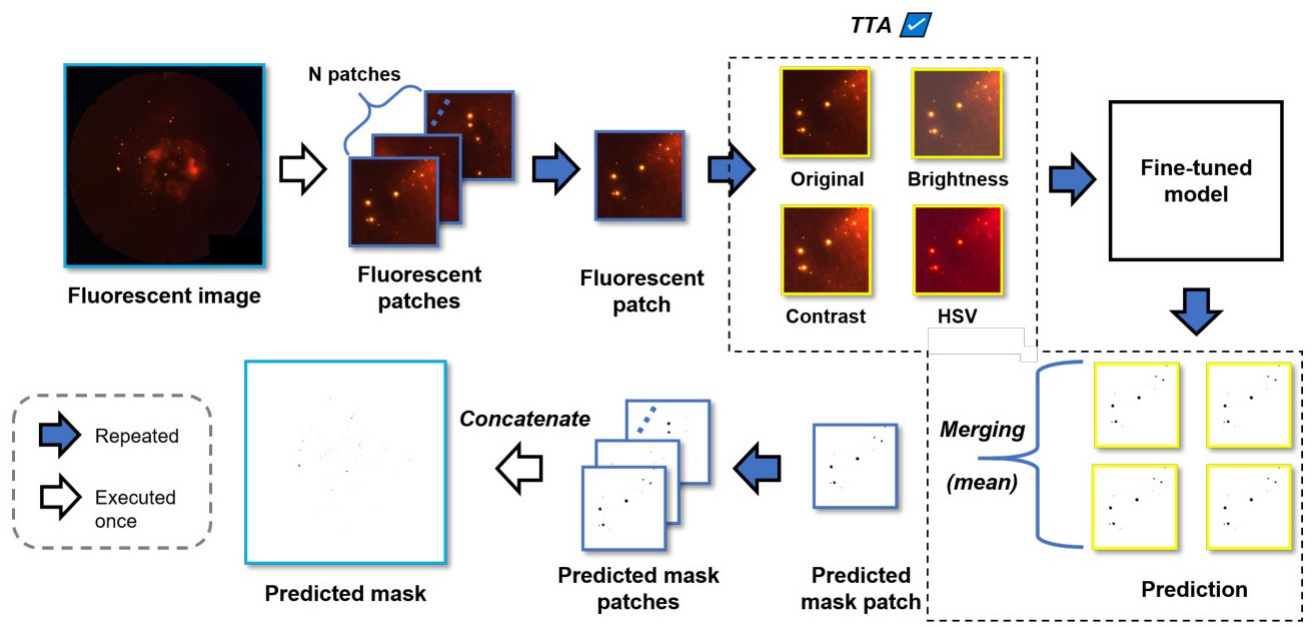
Mask generation for the fluorescence images in the set used for testing. The generated masks are compared with the ground truth masks to evaluate the effectiveness of a model, hereby using different metrics. When TTA is not implemented, the predicted mask patches are generated directly from the fluorescence patches. In contrast, when TTA is implemented (depicted with a dotted box), the fluorescence patches are augmented and the masks obtained for these patches are merged into a single mask by taking the pixel-wise average of each prediction.

The testing phase aims at determining the effectiveness of the models that have been created after fine-tuning. As shown in Fig 7, an input image *I* is split into small patches of the same size, and each patch is then fed into the fine-tuned model. This model subsequently predicts the corresponding mask patch. In the next step, the predicted mask patches are merged back into a predicted mask. The effectiveness of a model is evaluated by determining the similarity between the predicted mask and the ground truth mask. In this study, we also made use of TTA, which is a method that aims at increasing the prediction effectiveness by applying one or more augmentations to each patch. The augmented patches are then fed into the model to make multiple predictions. These predictions, including that of the original patch, are combined into a single prediction through simple pixel-wise averaging. A detailed explanation of TTA can be found in S4.1 Section in S4 File.

To determine the final effectiveness of the different models, we adopted five metrics commonly used in the field of computer vision, namely balanced accuracy, recall, precision, *F*_1_-score, and the Intersection-over-Union (IoU). A detailed description of each metric can be found in S4.2 Section in S4 File. Since the already existing TR-based models do not need fine-tuning, the final effectiveness, as measured by the different metrics, corresponds to the mean score of that metric over all images in the test set. On the other hand, since the TR-based deep learning models were fine-tuned using four different fold combinations, the final effectiveness was calculated by averaging the four means obtained for the different metrics (one mean per fine-tuned model). A lowercase letter m is added in front of each metric to signify that the numbers represent mean values.

### 4.4 Recovery assessment

To take into account the point-of-view of environmental scientists, we also evaluated the effectiveness of the best model in predicting MP count by assessing its recovery ability. This is done by calculating the ratio of the predicted MP count to the baseline MP count (as obtained for the ground truth mask), subsequently converting this ratio to a percentage. Percentage recovery is more relevant in the field of MP monitoring since the level of MP contamination is usually expressed as the number of MP per weight or volume of the sample under investigation [36]. In this respect, it should be clear that an accurate prediction of the MP count is of utmost importance.

For this purpose, five images containing fluorescent MP standards, which are microplastics of known composition (polymer type and average size), were created. These images are referred to as spiked images since microplastics were intentionally added (available at http://www.kaggle.com/sanghyeonaustinpark/mpset). The polymer types used were HDPE (high-density polyethylene; Dow^™^ HDPE KT 10000 UE), and PET (polyethylene terephthalate; Lighter^™^ C93 PET). The MP standards were prepared using a controlled milling process by the Center of Polymer and Material Technologies of the Department of Materials, Textiles and Chemical Engineering of Ghent University. HDPE (1 mg) and PET (0.5 mg) were suspended in 15 ml zinc chloride and stained following the procedure described in Section 3.1. Afterwards, images were captured and the masks were generated following the steps outlined in Section 3.2. These masks were then manually corrected through individual pixel annotation by three different researchers (see S1.1 Section in S1 File). Through a majority voting strategy, the final MP count was determined, which was used as the baseline (ground truth).

### 4.5 Statistical analysis

Student’s t-test was used to compare the mean positive pixels (MP pixels in a mask) between the ground truth and the different methods under investigation, which include the deep learning models and the already existing tools. The Bartlett test was performed prior to the Student’s t-test to validate equal or unequal variance between the ground truth and the predicted mask that is under consideration. In addition, ANOVA was applied to detect differences in the means of the number of TPs (true positives), FPs (false positives), TNs (true negatives), and FNs (false negatives) obtained from the masks generated by the different methods. A TP denotes an MP pixel correctly predicted as MP, an FP denotes a background pixel wrongly predicted as an MP pixel (Type 2 error), a TN refers to a background pixel correctly predicted as background, and an FN denotes an MP pixel wrongly predicted as a background pixel (Type 1 error). Failure to reject the null hypothesis would indicate that the mean number of either TPs, FPs, TNs, or FNs between different models is not significantly different.

## 5 Results and discussion

The ability of MP-VAT, MP-VAT 2.0, and C-VAT to predict the quantity of MP in a fluorescence image was first evaluated using the 99 images in Dataset B. These predicted MP quantities were compared to what we consider to be the true MP count derived from the masks, following the procedure described in S1.1 Section in S1 File.

**Fig 8:**
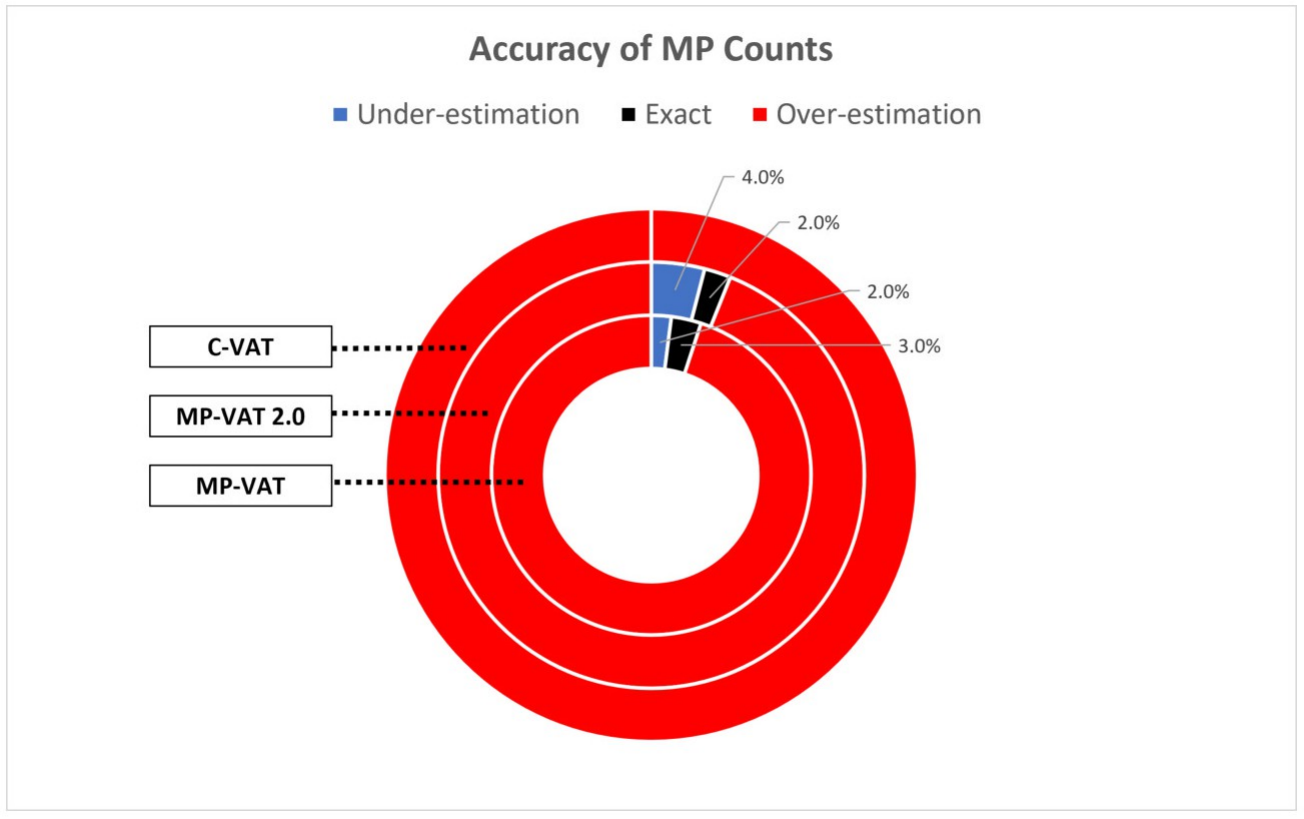
Accuracy of MP counts by MP-VAT, MP-VAT 2.0, and C-VAT. Predictions of MP quantity that are less than, equal to, and more than that of the ground truth are denoted as under-estimation, exact, and over-estimation, respectively. At least 94% of the predicted values from C-VAT, MP-VAT, and MP-VAT 2.0 were over-estimations.

Based on Fig 8, the predicted quantity of MP is frequently higher than the ground truth quantity when MP-VAT and MP-VAT 2.0 were used. In the case of C-VAT, all predictions were over-estimated. This mismatch between the binary masks created by the three existing methods and the fluorescence images is most likely caused by the use of global thresholding, as previously described in Section 2.2. Furthermore, the observed errors, particularly for MP-VAT and MP-VAT 2.0, may also be caused by background fluorescence and fluorescence halos belonging to very bright particles, as previously noted by the authors of [6].

In the following sections, we compare the effectiveness of the three already existing methods to the effectiveness of our deep learning models. We would like to emphasize that we first carried out experiments using U-Net (1)—(3), as previously discussed in [8]. After observing the good effectiveness of U-Net (3), as shown in Table 4, we decided to additionally investigate the effect of changing the optimizer from Adaptive Moment Estimation (Adam) to Stochastic Gradient Descent (SGD) [37, 38]. This new combination of Dice loss and SGD resulted in the creation of U-Net (4). Moreover, given that U-Net (3) and U-Net (4) are the most effective U-Net variations, we also decided to equip these models with TTA, as described in more detail in S4.1 Section in S4 File. Finally, we also experimented with a number of other contemporary models, namely FCN [30], DeepLabv3 [31], and Nested U-Net [32].

**Table 4:** Performance results obtained for Dataset B. The numbers between parentheses are standard deviations. The last column contains the p-values of the t-test comparing the mean MP pixel count between ground truth and predicted masks.

Model	Loss	Optimizer	mAccuracy	mRecall	mPrecision	m*F*_1_-score	mIoU	p-value
MP VAT	X	X	0.731 (0.056)	0.965 (0.111)	0.451 (0.212)	0.574 (0.218)	0.429 (0.190)	0.001
MP VAT 2.0	X	X	0.527 (0.161)	0.561 (0.317)	0.172 (0.248)	0.191 (0.199)	0.119 (0.131)	0.001
C-VAT	X	X	0.542 (0.174)	0.629 (0.383)	0.007 (0.021)	0.012 (0.035)	0.006 (0.019)	0.002
U-Net (1)	BCEWithLogits	SGD	**0.692 (0.013)**	**0.883 (0.026)**	0.517 (0.091)	0.601 (0.079)	0.466 (0.070)	0.278
U-Net (2)	DiceBCE	Adam	0.637 (0.011)	0.775 (0.022)	0.783 (0.022)	0.727 (0.004)	0.601 (0.004)	0.723
U-Net (3)	Dice	Adam	0.633 (0.011)	0.767 (0.021)	0.794 (0.013)	0.728 (0.002)	0.606 (0.004)	0.896
U-Net (4)	Dice	SGD	0.640 (0.020)	0.779 (0.040)	0.803 (0.041)	**0.736 (0.014)**	**0.617 (0.018)**	0.639
TTA U-Net (3)	Dice	Adam	0.597 (0.029)	0.694 (0.058)	**0.820 (0.055)**	0.696 (0.015)	0.562 (0.016)	0.607
TTA U-Net (4)	Dice	SGD	0.648 (0.026)	0.795 (0.052)	0.766 (0.072)	0.726 (0.017)	0.600 (0.024)	0.711
FCN	Dice	SGD	0.610 (0.020)	0.719 (0.039)	0.659 (0.024)	0.637 (0.012)	0.488 (0.009)	0.751
DeepLabv3	Dice	Adam	0.623 (0.021)	0.745 (0.042)	0.634 (0.030)	0.643 (0.008)	0.495 (0.009)	0.917
Nested U-Net	BCEWithLogits	Adam	0.675 (0.019)	0.851 (0.039)	0.710 (0.045)	0.725 (0.007)	0.597 (0.010)	0.556

### 5.1 MP and background classification (segmentation)

#### 5.1.1 Qualitative results

**Fig 9:**
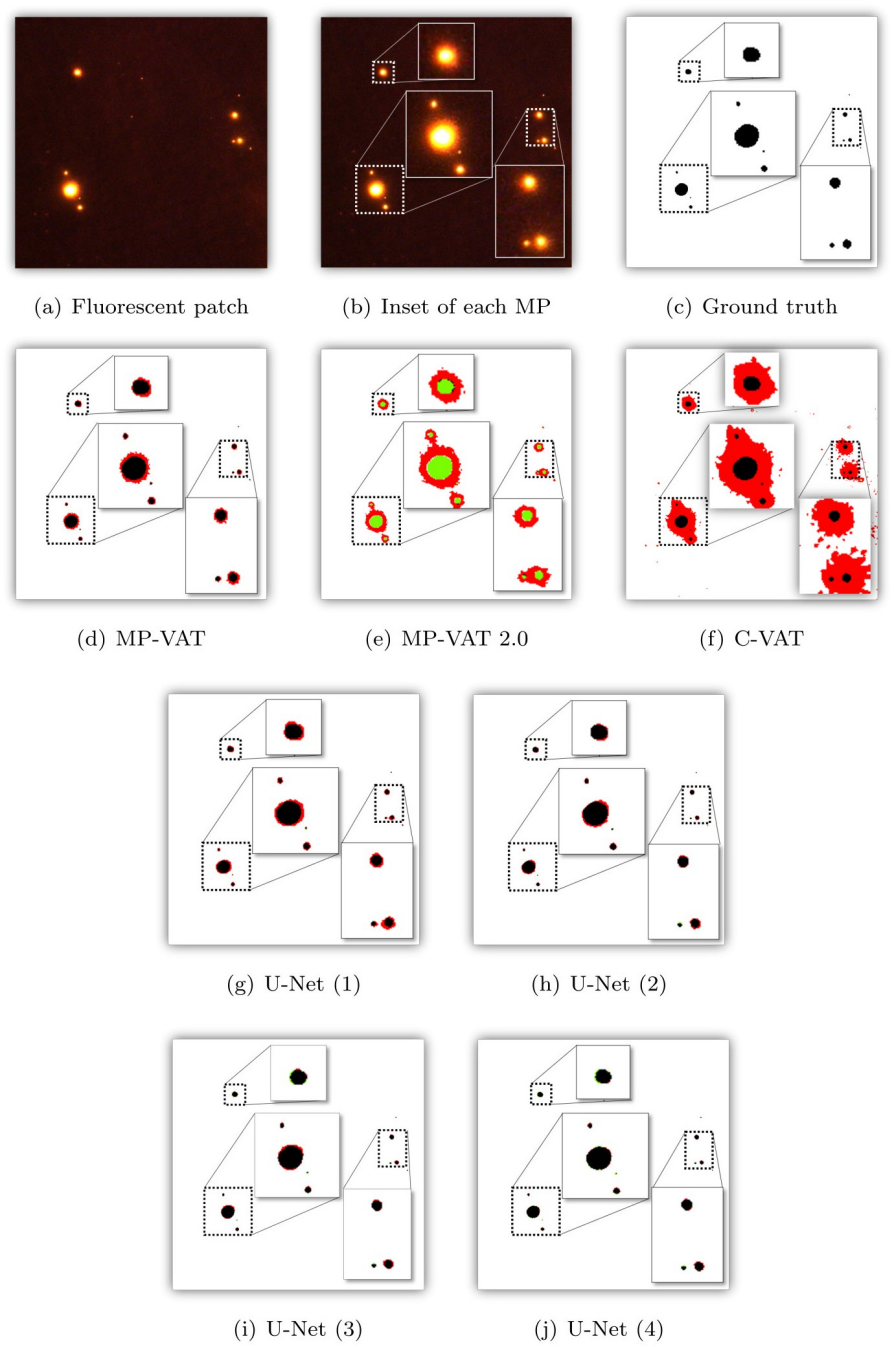
Visual comparison of the masks generated when making use of MP-VAT, MP-VAT 2.0, C-VAT, and the U-Net variations. For better visualization and understanding, regions with MP are also represented by making use of zoomed-in insets. Black denotes MP that has been correctly predicted (TP), red denotes predicted MP that is not MP (FP), and green denotes MP that has not been predicted as MP (FN). (a) Fluorescent patch. (b) Inset of each MP. (c) Ground truth. (d) MP-VAT. (e) MP-VAT 2.0. (f) C-VAT. (g) U-Net (1). (h) U-Net (2). (i) U-Net (3). (j) U-Net (4).

**Fig 10:**
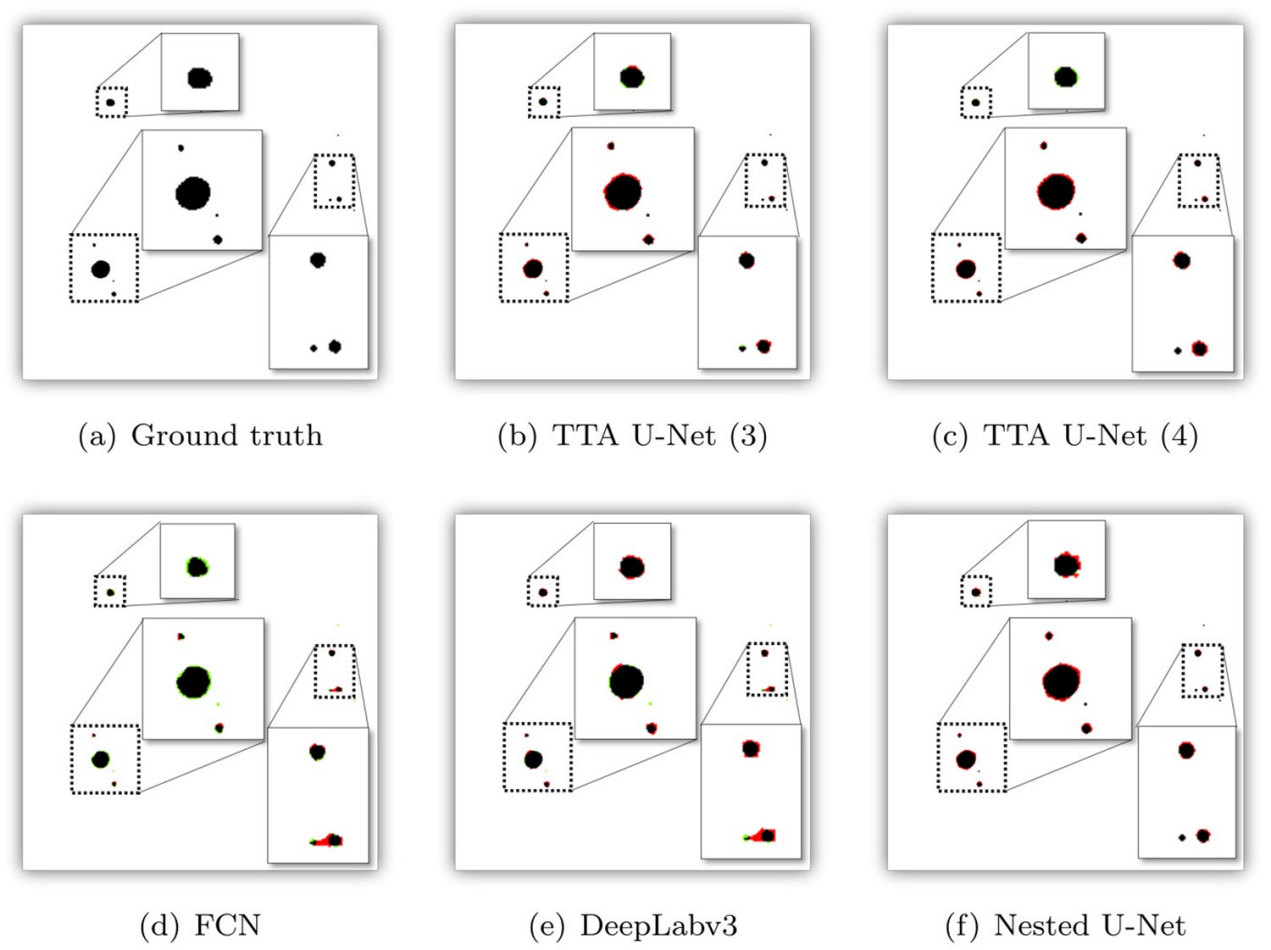
Visual comparison of the masks generated when making use of TTA, FCN, DeepLabv3, and Nested U-Net. For better visualization and understanding, regions with MP are also represented by making use of zoomed-in insets. Black denotes MP that has been correctly predicted (TP), red denotes predicted MP that is not MP (FP), and green denotes MP that has not been predicted as MP (FN). (a) Ground truth. (b) TTA U-Net (3). (c) TTA U-Net (4). (d) FCN. (e) DeepLabv3. (f) Nested U-Net.

Figs 9 and 10 visualize the differences in the binary masks generated by the different approaches used, relying on a representative patch taken from one of the images in the test set. In the different masks shown, the background pixels are white (TNs). The TPs, the FPs, and the FNs are colored black, red, and green, respectively.

As shown in Fig 9, it is evident that all deep learning models perform better than MP-VAT 2.0 and C-VAT. In particular, for the different U-Net variations, we observe a significant reduction in the number of FPs and the number of FNs. The same observation can be made for the masks generated by TTA U-Net (3), TTA U-Net (4), FCN, DeepLabv3, and Nested U-Net, as illustrated in Fig 10. Among the different models evaluated, U-Net (1) closely resembles MP-VAT and comes with the highest number of FPs. On the other hand, FCN generates the highest number of FNs. FCN and DeepLabv3 seem to be prone to errors caused by MP pieces that are in close proximity to each other. This can be seen in the lower right insets of Fig 10(d) and 10(e), showing how two MP pieces are merged into one. Based on the aforementioned observations, we can conclude that U-Net (1), FCN, and DeepLabv3 perform poorly compared to the other models.

Furthermore, we observe that only minute differences can be found between the masks generated by U-Net (2), U-Net (3), and Nested U-Net. On the other hand, U-Net (4) shows a substantial reduction in the number and the size of the FP regions. Although there are faint traces of visible FN regions, the mask created by U-Net (4) still closely resembles the ground truth mask, compared to the masks produced by U-Net (2), U-Net (3), and Nested U-Net. Moving to the TTA counterparts of U-Net (3) and U-Net (4), there seems to exist a subtle difference between U-Net (3) and TTA U-Net (3), as illustrated by the masks that can be found in Figs 9(h) and 10(b), respectively. In contrast, the mask produced by TTA U-Net (4), as shown in Fig 10(c), contains more FP regions compared to the mask produced by U-Net (4), as shown in Fig 9(i). In summary, U-Net (4) comes with the highest number of TPs and the lowest number of FPs and FNs, making it the best performing model in qualitative terms.

#### 5.1.2 Quantitative results

Table 4 shows the quantitative results obtained for Dataset B, as described in detail in S1.1 and S1.2 Sections in S1 File.

A comparison of the mean MP pixel count between the ground truth and the predicted masks was performed for the three existing methods and the TR-based deep learning models. A significantly higher mean pixel count was observed for MP-VAT, MP-VAT 2.0, and for C-VAT, as shown in the last column of Table 4. This result agrees with the previous observations that these three existing tools overestimate the quantity of MP. On the other hand, none of the predictions made by the deep learning models showed significant differences from the ground truth, suggesting that, in this respect, these models perform better than already existing tools. When the mean values of pixels corresponding to TPs, FPs, TNs, and FNs were compared through ANOVA, both mean FP and mean TN were found to be considerably different (*p* < 0.05) between MP-VAT, MP-VAT 2.0, C-VAT, and each of the deep learning models (the detailed numbers can be found in the S1 Table). Based on this observation, a reduction in the number of FPs and subsequent improved recognition of TNs seems to have been achieved by the deep learning models, thereby leading to MP quantity predictions that are similar to the ground truth. Among the nine TR-based deep learning models, U-Net (1) showed the highest effectiveness in terms of mAccuracy (0.692) and mRecall (0.883). On the other hand, TTA U-Net (3) obtained the highest mPrecision (0.820), whereas U-Net (4) achieved the highest m*F*_1_-score (0.736) and the highest mIoU (0.617). Given these observations, we conclude that the most effective models are U-Net (1), TTA U-Net (3), and U-Net (4), in no particular order. In order to select the most effective model, we decided to attach more importance to metrics that appropriately capture the concept of distinguishing MP, which is a sparse class, from other image elements. In this respect, the metrics highly influenced by TP were considered to be relatively more significant.

As shown in S4.2 Section in S4 File, mAccuracy is highly affected by TN. Indeed, since TN corresponds to correctly identified background, its value is substantially higher than the value of TP. For this reason, mAccuracy was considered to be a metric of less importance. On a similar note, both mRecall and mPrecision assign significant weight to TP. However, these two metrics only account for either FP or FN. Because of this, mRecall and mPrecision were also considered to be of less importance. Furthermore, m*F*_1_-score and mIoU comprehensively take into account TP, FP, and FN. In addition, they are not affected by TN, thereby making them the most suitable metrics to determine the most effective model. As a result, U-Net (4), which showed the highest m*F*_1_-score and mIoU, was chosen as the best TR-based deep learning model. This choice is also supported by the mask patches presented in Figs 9 and 10. As discussed in Section 5.1.1, the masks generated by U-Net (4) resemble the ground truth masks more closely, compared to the masks produced by other models. Hence, U-Net (4), which is referred to as MP-Net throughout the remainder of the paper, exhibited the best performance, both quantitatively and qualitatively.

Additional experimental results such as assessment of the use of different encoders, analysis of the impact of TTA, and an ablation study on different types of augmentation, can be found in S6.1, S6.2 and S6.4 Sections in S5 File, respectively.

### 5.2 Recovery assessment

Recovery assessment was performed using MP-Net, which was found to be the best TR-based deep learning model. This type of assessment was conducted using two sets of images with a different level of complexity: (1) a first set of five spiked images containing only MP standards and (2) a second set of 15 real images of MP extracted from clams. In the spiked images (available at http://www.kaggle.com/sanghyeonaustinpark/mpset), the MP standards have less variability in particle size and shape since they were produced through a controlled milling process. On the other hand, the real images contain MP with a varying size and shape since they were extracted from environmental samples, specifically from clams, thus adding complexity to the images. Due to the limited availability of MP standards, only five spiked images were created for recovery assessment.

As shown in Table 5, the different versions of MP-VAT obtain extremely high percentage recoveries for the spiked images, with a minimum of 169% and a maximum of 299%. Similarly, C-VAT shows percentage recoveries of more than 158%. This translates to detecting two to three times the actual MP quantity. On the other hand, MP-Net shows percentage recoveries close to 100% for the majority of the spiked images, obtaining an average of 107.8±9.2%.

**Table 5:** MP count and percentage recovery obtained for five spiked MP images. The MP quantity predicted by MP-Net was much closer to the ground truth compared to MP-VAT, MP-VAT 2.0, and C-VAT.

Sample number	Ground truth (Majority vote)	MP-VAT		MP-VAT 2.0		C-VAT		MP-Net	
		MP count	Percentage recovery	MP count	Percentage recovery	MP count	Percentage recovery	MP count	Percentage recovery
1	170	287	169	396	233	268	158	175	103
2	91	163	179	204	224	166	182	100	110
3	132	337	255	389	295	353	267	135	102
4	53	122	230	153	289	128	242	65	123
5	131	258	197	392	299	284	217	132	101
Average (Standard deviation)			206.0 (35.9)		268.0 (36.4)		213.2 (36.03)		107.8 (9.2)

When it comes to real images, the different versions of MP-VAT have even more extreme percentage recoveries, as demonstrated in Table 6. Significantly high values of 3,200% and 5,940% were observed for MP-VAT and MP-VAT 2.0, respectively. C-VAT exhibits even higher percentage recoveries, reaching values of up to 57,020%. For MP-Net, overestimation was also observed but to a lesser extent, with a maximum percentage recovery of 150%. Some cases of underestimation were also detected. For instance, samples 5 and 8 have percentage recoveries of only 83% and 77%, respectively. Despite these high and low percentage recoveries obtained by MP-Net for real images, its output is still the closest to the ground truth, with an average percentage recovery of 107.5±21.0%.

**Table 6:** MP count and percentage recovery obtained for 15 clam MP images. The MP quantity predicted by MP-Net was much closer to the ground truth compared to MP-VAT, MP-VAT 2.0, and C-VAT.

Sample number	Ground truth (Majority vote)	MP-VAT		MP-VAT 2.0		C-VAT		MP-Net	
		MP count	Percentage recovery	MP count	Percentage recovery	MP count	Percentage recovery	MP count	Percentage recovery
1	19	29	153	602	3,168	2,951	15,531	17	89
2	5	19	380	21	420	2,851	57,020	6	120
3	8	11	138	14	175	185	2,312	8	100
4	6	7	117	326	5,433	256	4,266	5	83
5	7	8	114	18	257	355	5,071	6	86
6	5	16	320	297	5,940	15	300	7	140
7	383	655	171	609	159	46,066	12,027	294	77
8	2	64	3,200	9	450	283	14,150	2	100
9	4	39	975	34	850	26	650	6	150
10	223	564	253	449	201	22,819	10,232	230	103
11	11	38	345	28	255	653	5,936	13	118
12	26	42	162	49	188	261	1,003	27	104
13	74	268	362	138	186	5,378	7,267	92	124
14	94	294	313	3,145	3,346	20,943	22,279	114	121
15	138	288	209	1,301	943	2,304	1,669	135	98
Average (Standard deviation)			480.8 (781.5)		1,467.7 (2,002.5)		10,647 (13,415.1)		107.5 (21.0)

**Fig 11:**
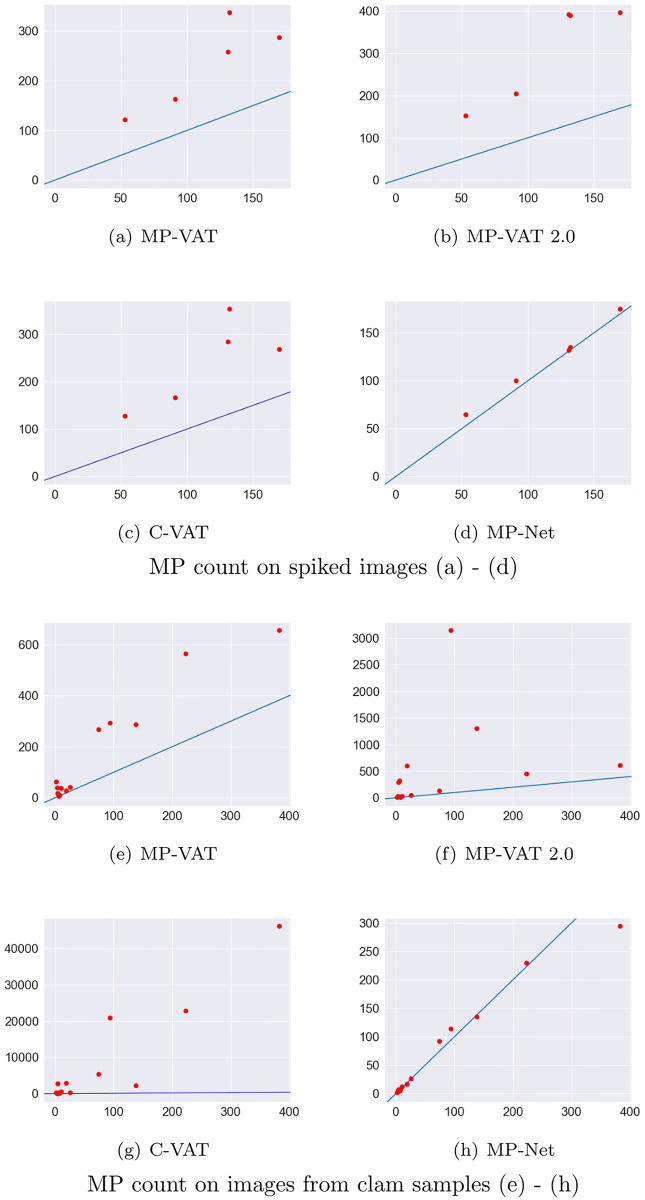
Recovery assessment. Comparison of MP counts obtained for five spiked images (a)—(d) and 15 real images from clam samples (e)—(h). Red dots represent tested images. The x-coordinate refers to the ground truth MP count and the y-coordinate denotes the predicted MP count. For a red dot above the first bisector (blue line), the number of MPs is overestimated by the model (MP-VAT, MP-VAT 2.0, C-VAT and MP-Net). (a) MP-VAT. (b) MP-VAT 2.0. (c) C-VAT. (d) MP-Net. (e) MP-VAT. (f) MP-VAT. (g) C-VAT. (h) MP-Net. MP count on spiked images (a)–(d). MP count on images from clam samples (e)–(h).

Overall, the different versions of MP-VAT and C-VAT consistently produce over-inflated estimates of the MP quantity in both spiked and real images. As shown in Fig 11(a)–11(c) and 11e–11(g), the predicted values fall far from the ground truth, which is depicted by the blue line. On the other hand, predictions from MP-Net lie closer to the blue line, as illustrated in Fig 11(d) and 11(h).

## 6 Conclusions

In this study, we proposed and evaluated several computational approaches towards MP quantification. To that end, we created a set of fluorescence microscopy images of MP, together with corresponding binary masks. This was done by first extracting MP from clams and by subsequently applying Nile red staining, making it possible to highlight MP debris under a microscope. Through a thorough survey of the literature, we identified already existing semi- or fully automated tools used to analyze MP. Applying three of these tools, namely MP-VAT, MP-VAT 2.0, and C-VAT, to our set of MP images showed that the use of a global TR-based method results in MP overestimation and mislabeling. We hypothesized that these weaknesses could be overcome by leveraging a TR-based deep learning model, with this end-to-end learning approach being able to mimic the way researchers identify MP in given fluorescence microscopy images.

Next to MP-VAT, MP-VAT 2.0, and C-VAT, we tested and compared a total of nine deep learning models through common performance metrics and visual inspection of the resulting mask patches. U-Net (4), which we named MP-Net, was found to be the most effective in terms of MP segmentation. Specifically, MP-Net exhibited the highest m*F*_1_-score and mIoU. Furthermore, we found the masks generated by MP-Net to be the most consistent with the ground truth masks. Recovery assessment using both spiked and real images also demonstrated that MP-Net produces better predictions of MP count than the three already existing tools. Indeed, even though slightly under- and overestimated MP counts were observed for MP-Net, the percentage recoveries were much closer to 100%, compared to the percentage recoveries obtained by MP-VAT, MP-VAT 2.0, and C-VAT.

As part of the study presented in this manuscript, we make available MAP, which stands for Microplastics Annotation Package, an integrated MP annotation tool equipped with a user-friendly graphical user interface. Specifically, MAP incorporates MP-Net, the first TR-based deep learning model for MP detection, and the different TR-based models shown in Table 2. For each piece of MP found in a mask (MC), MAP also creates a summary table containing SM and SC. Furthermore, MAP provides options for manual annotation and model fine-tuning, thereby allowing a model to be tailored to the specific needs of researchers.

Through this study, we would like to encourage researchers in the field of computer vision to consider expanding their field of research with the topic of MP analysis. To that end, all the code used in our experiments is made publicly available, together with a representative set of patches obtained from real images (see Section 8.2). The five spiked images used in our recovery assessment experiment are also released, together with the bright-field microscopy images and the corresponding masks (see Section 8.3).

## 7 Directions for future work

### 7.1 Improving segmentation effectiveness

#### 7.1.1 Investigating encoder and augmentation combinations

Looking at the additional experimental results provided in S6.1 and S6.2 Sections in S5 File, we believe it is possible to still find a better model than the already existing ones. In particular, although the performance evaluation presented in S6.1 and S6.2 Sections in S5 File was conducted only for Fine-tuning (1) (see Fig 5), the use of different encoder and augmentation combinations may result in a higher effectiveness than that of MP-Net. Indeed, as illustrated in S6.1 Section in S5 File, the use of a ResNet-18 encoder or a ResNet-34 encoder allows for a higher effectiveness than the use of a ResNet-101 encoder. In addition, augmentation by combining random brightness and random contrast allows achieving the highest values in terms of mAccuracy, mRecall, m*F*_1_-score, and mIoU, as shown in S6.3 Section in S5 File. Considering these encoder and augmentation combinations at the same time, it may be possible to obtain a TR-based deep learning model that is even better performing. However, more extensive testing is needed to confirm this hypothesis.

#### 7.1.2 Use of generative adversarial networks

As a candidate technique for building TR-based deep learning models, it may be of interest to consider the adoption of Generative Adversarial Networks (GANs). A GAN is a type of deep learning model that was introduced in [39]. If we see a conventional deep learning model as a single model that learns the relationship between input and ground truth pairs, making predictions close to the ground truth, then GANs can be seen as structures that are composed of two models, namely a generator and a discriminator. Assuming a computer vision use case, the generator receives a random number and generates a fake input image. The discriminator then determines whether the input image is real or fake. When these two models go through a series of training steps, the generator learns to produce more plausible images. In other words, the generator learns to understand the desired image characteristics.

Starting from the MNIST dataset [40], GANs have been gradually applied to computer vision problems. For instance, [41, 42] present semi-supervised classification approaches using GANs. Furthermore, Xue et al. [43], Zhang et al. [44], and Majurski et al. [45] are addressing image segmentation problems using GANs.

Pix2pix [46], which uses conditional GANs, may be of particular interest. Here, a generator takes as input an edge outline, a map, or a mask and then produces an output image corresponding to the given input. The following use cases are suggested by the authors: (1) input: satellite image, output: corresponding map image and (2) input: a silhouette image of an object (e.g., a pair of shoes), output: an image containing the corresponding object. If such a model receives a fluorescence microscopy image as input and subsequently outputs a corresponding MP mask, then the effectiveness of this model can be evaluated and compared with other deep learning-based approaches.

#### 7.1.3 Use of active learning (human-in-the-loop)

It may be of interest to incorporate a tool from the field of artificial intelligence that is commonly referred to as Active Learning [47] or Suggestive Annotation. Specifically, using this tool, it is possible to calculate the uncertainty of each pixel in a given image and to actively suggest regions of high uncertainty for researchers to examine and decide upon. That way, the model is able to learn the annotation strategy used by researchers, leading to more accurate annotations.

### 7.2 Upgrading MAP

Our annotation software can be further improved in three ways.

First, it would be of interest to implement the RGB thresholding method of ImageJ. Unlike already existing TR-based models that use a single threshold value, ImageJ has an option for distinguishing MP pixels from background pixels by setting six thresholds, namely a minimum and maximum threshold for each RGB channel. By incorporating this approach, users will be able to proceed with MP monitoring in a more flexible way.

**Fig 12:**
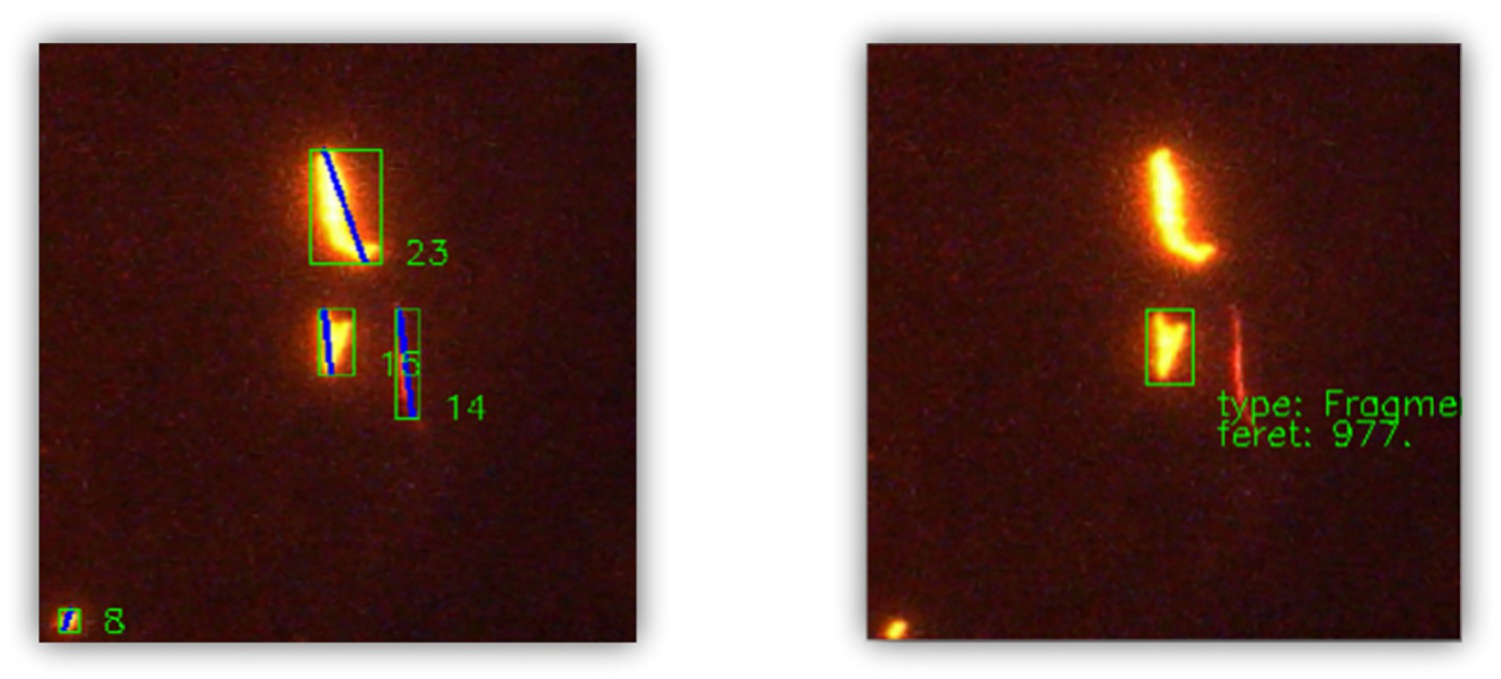
Representative patches illustrating MP visualization in MAP.

Second, it would be of interest to have support for individual MP visualization. Currently, it is not possible to highlight a particular piece of MP in an image. Instead, all detected MP are highlighted and enclosed in a box as shown in Fig 12(a). This can be improved by adding a function that highlights a selected piece of MP, as shown in Fig 12(b), providing detailed visual information regarding its size and shape. That way, it is possible to generate better visualizations for images having a high MP density. In relation to this, filtered visualizations could also be incorporated where only MP with a specific shape or a particular size range are highlighted. In that manner, the annotation software becomes more interactive.

Third, it would be of interest to have support for batch processing and batch reports. Usually, MP monitoring studies generate one fluorescence image per sample. Each image is then individually analyzed using the desired TR-based method, resulting in the generation of a file containing MC, SM, and SC for each piece of MP found in the given image. This means that, when there are multiple images, an identical number of files will be produced. Researchers have to collect and organize these files in order to analyze and synthesize the data generated. To assist researchers in this task, we plan to add a function to MAP that provides batch processing of multiple images, followed by the generation of an integrated statistical report containing summarizing charts and tables, thus making it possible to reduce the amount of time spent by researchers on data curation.

## 8 Software and dataset availability

### 8.1 MAP: A GUI-based annotation tool

- Software name: Microplastics Annotation Package (MAP)
- Hardware & OS requirements: PC (Windows, Linux, Mac)
- Year of first official release: 2021
- Code size: 8 MB (+35 ∼ 233 MB for model parameters)
- Availability: https://github.com/powersimmani/Microplastics-Annotation-Package
- License: GPL 3.0
- Documentation: README in GitHub repository
- Video tutorial: https://www.youtube.com/watch?v=ehmRbTbqOKU

### 8.2 MP-Net: Training and evaluation code for MP segmentation

- Software name: MP-Net
- Hardware & OS requirements: PC (Windows, Linux, Mac)
- Year of first official release: 2021
- Code size: 8 MB (+35 ∼ 233 MB for model parameters)
- Availability: https://github.com/sanghyeonp/MP-Net
- License: MIT 3.0
- Documentation: README in GitHub repository

### 8.3 MP-Set: Fluorescence image and mask pairs used to obtain the experimental results

- Dataset name: MP-Set
- Year of first official release: 2022
- Dataset size: 2.25 GB
- Availability: www.kaggle.com/sanghyeonaustinpark/mpset
- License: CC-BY 4.0
- Documentation: README in repository

## Supporting information

S1 File Dataset preparation. (PDF)

S2 File Fine-tuning phase. (PDF)

S3 File Learning curves. (PDF)

S4 File Testing phase. (PDF)

S5 File Additional results. (PDF)

S1 Table Statistical analysis. (XLSX)
