# Supplementary material for: MP-Net: Deep learning-based segmentation for fluorescence microscopy images of microplastics isolated from clams
Source: PLoS One. 2022 Jun 15;17(6):e0269449. doi: 10.1371/journal.pone.0269449 (PMC9200300; doi:10.1371/journal.pone.0269449)
Supplement: S1 File — (PDF) [file pone.0269449.s001.pdf]

S1. Dataset preparation

S1.1 Dataset annotation

As explained in (main text) Figure 1 (a), it is difficult to accurately label all MP when making use of a single threshold. Figure 1 (b) shows an example of an incorrect prediction (Type 2 error), and similar incorrect predictions have also been reported in [1]. Since uncertain labeling may have a significant impact on the effectiveness of TR-based deep learning models, we improved the quality of our labels by incorporating individual pixel annotation (IPA), as shown in Figure 2 (b).

Although doing so required a substantial amount of manual effort, it was a necessary step to ensure that our TR-based models are trained with properly annotated images. To avoid bias, three researchers participated in the annotation process. Annotation was completed through Microsoft Paint and Medibang Paint.[1] The final mask was obtained using a majority voting strategy, following the opinion of the majority of the annotators.

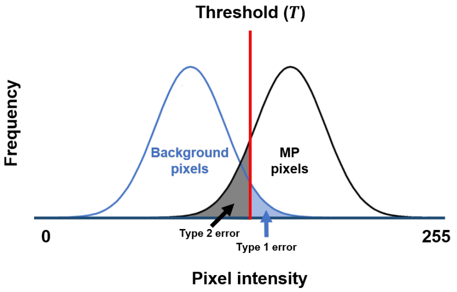

(a) Classification of MP according to a particular threshold value T. The x-axis denotes pixel intensity and the y-axis denotes the number of pixels having the corresponding pixel intensity value. Blue indicates the distribution obtained for the background pixels and black indicates the distribution obtained for the MP pixels. Errors introduced by staining and capturing MP create an overlapping area of pixels that are difficult to categorize.

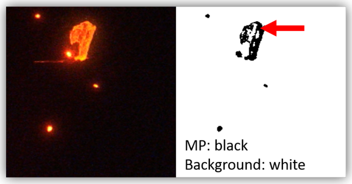

(b) An example of a Type 2 error. The pixels the red arrow points to appear as MP pixels on the fluorescence microscopy image, but are classified as background pixels because they are relatively darker than the other neighbouring MP pixels.

Figure 1: Type 1 and Type 2 errors when using a single value for thresholding.

S1.2 Image patch extraction using a sliding window approach and over-sampling

A deep neural network usually comes with a significant number of parameters. As a result, model training typically requires a substantial amount of data. However, our dataset for training only contains a total of 80 images. If we

[1] https://medibangpaint.com/

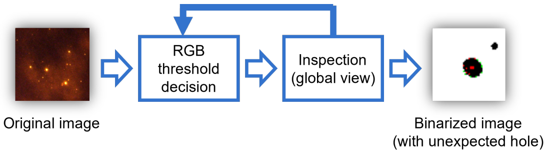

(a) RGB thresholding, taking 10 $\sim$ 15 min for labeling a single image. This approach was used to create Dataset A.

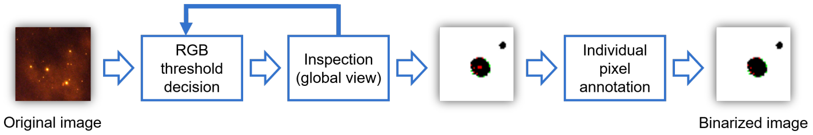

(b) RGB thresholding and individual pixel annotation. This approach was used to create Dataset B.

Figure 2: Overall MP annotation approach: (a) for Dataset A and (b) for Dataset B.

would simply slice each image into patches of 256×256 pixels, we would be able to obtain 15,983 patches. However, among these 15,983 patches, only 2,123 patches contain at least one piece of MP. Therefore, MP can be identified only in 13.3% of patches. To overcome the lack of available data and to mitigate the imbalance between the number of MP and background pixels, we applied two methods to create a dataset that is more suitable for the purpose of training: a sliding window and over-sampling.

First, as shown in Figure 3, overlapping patches were cropped from each image, using a stride of 30 pixels. Since cropping similar patches multiple times may cause overfitting, transfer learning was used, as discussed in Supporting information S2.5. Second, among the sampled patches, we removed patches without MP, thus implementing a form of over-sampling, which is a commonly used method in the field of machine learning to solve the problem of class imbalance [2]. Dataset B was created using this methodology and contains no patches without MP. For Dataset A, on the other hand, 5% of the patches do not have any MP.

S1.3 Under- and overestimation of MP count by the different MP-VAT versions

The MP count in the 99 fluorescence images in Dataset B was estimated using MP-VAT, MP-VAT 2.0, and C-VAT. These predicted MP quantities were compared to what we consider to be the true MP count, derived from the masks following the procedure described in Supporting information S1.1. The error between the predicted counts and the true counts was calculated according to the following formula:

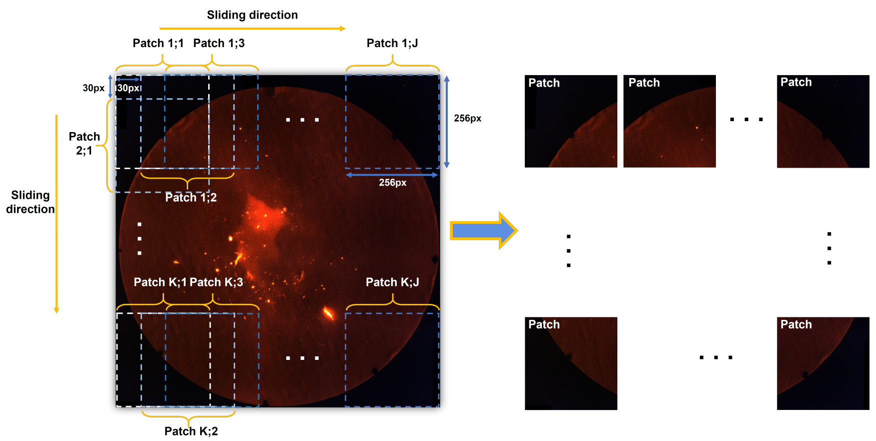

Figure 3: Patches of size $p \in \mathbb{R}^{256\times256}$ are generated by making use of a sliding window approach, applying a stride of 30 pixels. M and N represent the number of patches generated along the width and the height of an image, respectively.

$$\text{Percentage error} = \frac{\text{Predicted count} - \text{True count}}{\text{True count}} \times 100\,. \tag{1}$$

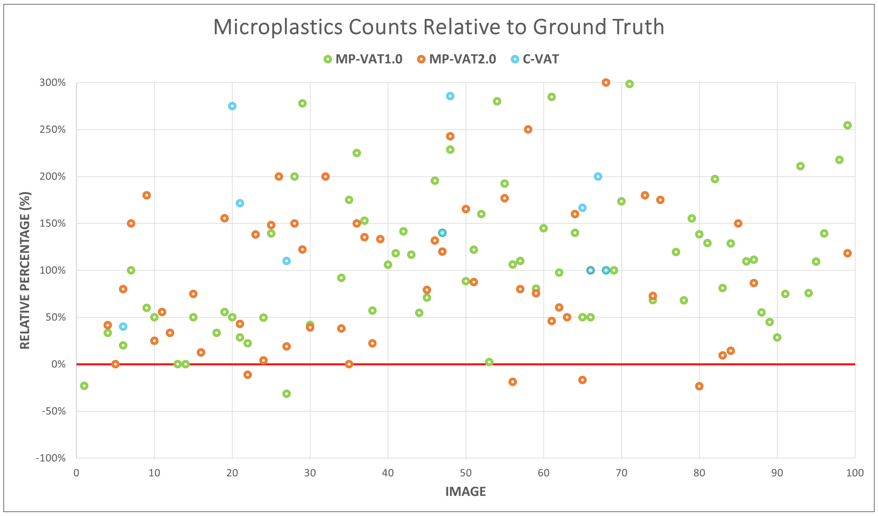

Figure 4: Scatter plot showing the percentage error in MP count predicted by MP-VAT, MP-VAT 2.0, and C-VAT. At 0% error, as depicted by the red line, the predicted count is equal to the true count. Above and below the red line, the predicted count is more than and less than the true count, respectively. Predictions with an error higher than 300% are not shown for clarity.

References

[1] Lorenzo-Navarro J, Castrillón-Santana M, Sánchez-Nielsen E, Zarco B, Herrera A, Martínez I, Gómez M. Deep learning approach for automatic microplastics counting and classification. Science of The Total Environment. 2021;765:142728. doi:10.1016/j.scitotenv.2020.142728.

[2] Buda M, Maki A, Mazurowski MA. A systematic study of the class imbalance problem in convolutional neural networks. Neural Networks. 2018;106:249–259. doi:10.1016/j.neunet.2018.07.011.
