# Supplementary material for: MP-Net: Deep learning-based segmentation for fluorescence microscopy images of microplastics isolated from clams
Source: PLoS One. 2022 Jun 15;17(6):e0269449. doi: 10.1371/journal.pone.0269449 (PMC9200300; doi:10.1371/journal.pone.0269449)
Supplement: S2 File — (PDF) [file pone.0269449.s002.pdf]

S2. Fine-tuning phase

S2.1 Overall summary

Table 1 shows the encoder, pre-training, and hyper-parameter settings used by the different experiments performed. In this study, a ResNet-101 [1] encoder was adopted as the feature extractor for all models. To prevent overfitting and to reduce the amount of time needed for training, the parameter values obtained through pre-training with ImageNet [2] or MS COCO 2017 [3] were transferred to the encoder and used for fine-tuning.

In the fine-tuning stage, the model predicted 10 mask patches at a time and compared those predictions to the corresponding 10 ground truth patches. After that, the model parameters were updated using the average value of the 10 losses. A mini-batch size of 10 was found to be the maximum value for which the available GPU memory capacity (8 GB) was not exceeded.

The TR-based deep learning models gradually reduce the training loss that occurs in the fine-tuning CVFs by performing multiple epochs. In each epoch, mini-batches were fed to a model until every patch in the fine-tuning CVFs was used once. At the end of every epoch, the validation CVF was used to obtain the validation loss and validation effectiveness of the model under consideration.

At the end of an epoch, the parameters of each model were only kept if the validation loss of the current epoch was less than the lowest validation loss found during the previous epochs. For U-Net, TTA U-Net, and Nested U-Net, this process was repeated for 20 epochs; for FCN and DeepLabv3, only 10 epochs were used since the optimization time for these models was more than double that of the others. The stored parameters were then used to calculate the final model performance for the test set.

More details about the models used in our experiments can be found in Supporting information S2.2. Furthermore, information about the loss functions and the optimizers used can be found in Supporting information S2.3 and Supporting information S2.4, respectively. Finally, transfer leaning using pre-trained parameters is explained in Supporting information S2.5.

Table 1: Encoder, pre-training, and hyper-parameter settings used by the experiments performed.

Model	Encoder	Dataset for pre-training	Loss		Optimizer		
			Type	Positive weight	Type	Learning rate	Momentum
U-Net (1)	ResNet-101	ImageNet	BCE With Logits	9	SGD	0.001	0.9
U-Net (2)	ResNet-101	ImageNet	Dice BCE	-	Adam	0.001	-
U-Net (3)	ResNet-101	ImageNet	Dice	-	Adam	0.001	-
U-Net (4)	ResNet-101	ImageNet	Dice	-	SGD	0.001	0.9
TTA U-Net (3)	ResNet-101	ImageNet	Dice	-	Adam	0.001	-
TTA U-Net (4)	ResNet-101	ImageNet	Dice	-	SGD	0.001	0.9
FCN	ResNet-101	MS COCO 2017	Dice	-	SGD	0.001	0.9
DeepLabv3	ResNet-101	MS COCO 2017	Dice	-	Adam	0.001	-
Nested U-Net	ResNet-101	ImageNet	BCE With Logits	9	Adam	0.001	-

S2.2 TR-based deep learning models for segmentation

S2.2.1 U-Net

We used U-Net [4], a popular convolutional neural network, as our basic segmentation model $\mathcal{M}$. In particular, U-Net is an encoder-decoder model that was initially developed for medical image segmentation. However, this model is currently also widely used outside the medical field [5].

The architecture of U-Net, as shown in Figure 1, consists of a contracting and an expansive path. The contracting path is composed of a series of convolutional layers that extract context from the input image. The final feature map obtained from the contracting path is deconvoluted in the expansive path. The deconvoluted feature map is then concatenated with a corresponding feature map from the contracting path through skip connections (grey arrows in Figure 1) for more precise localization in the image to be segmented.

For our experiments, we chose a U-Net model with a ResNet-101 [1] encoder, pre-trained on ImageNet [2]. A high-level Application Programming Interface (API) [6] has been used to build our segmentation models.

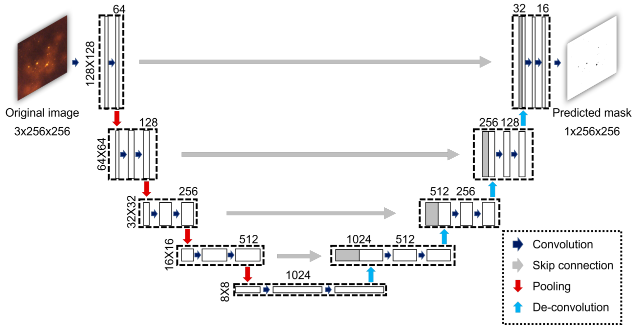

Figure 1: U-Net architecture [4]

.

S2.2.2 Other segmentation networks

Fully Convolutional Network (FCN) [7] proposed FCN, a CNN-based segmentation model. The defining characteristic of this model is that it consists only of convolutional layers and that it uses up-sampling. Furthermore, FCN does not divide an input image into small patches, but takes the whole image as input. However, for the sake of comparability, our FCN-based model was fine-tuned on a patch basis in our study. In addition, our FCN-based model used a ResNet-101 encoder with parameters pre-trained on MS COCO 2017 [3].

DeepLabv3 The DeepLab segmentation models collectively refer to models that use atrous convolution for dense feature extraction. Compared to former versions [8], DeepLabv3 [9] is inspired by ResNet [1], adopting the concept of

a residual block and deepening the feature extraction layers. In addition, the effectiveness of segmentation was improved by applying Atrous Spatial Pyramid Pooling, which explores convolutional features for various resolutions. Similar to our FCN-based model, our DeepLabv3-based model used a ResNet-101 encoder, applying fine-tuning to parameter values that have been pre-trained on MS COCO 2017 [3].

Nested U-Net (U-Net++) [10] proposed Nested U-Net (U-Net++), a neural network inspired by the dense block concept of DenseNet [11], improving upon U-Net by changing its structure. In particular, compared to U-Net, Nested U-Net has three major differences. The first difference is the use of convolutional skip connections, which aim at learning the semantic gap between encoder and decoder feature maps. The second difference is the use of dense skip connections, which improve the flow of gradients. Finally, by applying deep supervision, the model can be trained either (1) by using the entire model structure (better effectiveness) or (2) by pruning some layers (faster prediction speed). We used a pre-defined Nested U-Net architecture, as available from Yakubovskiy [6]

S2.3 Loss functions

In our experiments, the three loss functions discussed below were used. For this section, M refers to a mini-batch of ground truth masks and $\hat{M}$ refers to a mini-batch of predicted masks. On the other hand, M_l pertains to a single ground truth mask while $\hat{M}_l$ refers to a single predicted mask.

Binary cross-entropy (BCE) with logits loss BCE with logits loss is a modification of the regular BCE loss function [12], combining the sigmoid activation function and the BCE loss function into a single layer. The use of BCE with logits loss is numerically more stable than the independent use of the sigmoid activation function and the BCE loss function. Besides, a hyperparameter ϱ, called the positive weight, is introduced, making it possible to weight the positive samples in order to mitigate severe class imbalance. BCE with logits loss is defined as follows:

$$
\begin{aligned}
L_{BCE-L}(\hat{M}, M) = -\frac{1}{N} \sum_{l=1}^{N} \Big(& \varrho \cdot M_l \cdot log(\sigma(\hat{M}_l)) \\
& +(1 - M_l) \cdot log(1 - \sigma(\hat{M}_l)) \Big) ,
\end{aligned} \tag{1}
$$

where N represents the size of the mini-batch of images, ϱ represents the positive weight (set to 9), and σ represents the sigmoid activation function.

Dice loss The Dice coefficient is a measure for the overlap between a predicted segmentation mask and a ground truth segmentation mask, and is widely used for evaluating the effectiveness of segmentation models [13]. This measure was

used as a loss function, where this loss function is insensitive to data imbalance [14]. The Dice loss function is defined as follows:

$$L_{Dice}(\hat{M}, M) = \frac{1}{N}\sum_{l=1}^{N}\Big(1 - \frac{(2 \cdot M_l \cdot \hat{M}_l) + \varepsilon}{M_l + \hat{M}_l + \varepsilon}\Big)\,, \tag{2}$$

where ε allows avoidance of numerical issues when $M_l = \hat{M}_l = 0$. In our case, ε was set to 1.

BCE with Dice loss As discussed in [15], BCE with logits loss and Dice loss can be combined. The Dice loss focuses on the similarity between the prediction and the ground truth at the image level, whereas the BCE loss focuses on the pixel-wise differences between the prediction and the ground truth. BCE with Dice loss is defined as follows:

$$\begin{aligned} L_{BCE-D}(\hat{M}, M) = \frac{1}{N}\sum_{l=1}^{N}\Big(&L_{BCE-L}(\hat{M}_l, M_l) \\ &+ L_{Dice}(\hat{M}_l, M_l)\Big)\,. \end{aligned} \tag{3}$$

S2.4 Optimization methods

Gradient descent is used to update the parameters θ of our segmentation models in the negative direction of the gradient of an objective function $\nabla_\theta J(\theta)$, with the goal of minimizing the objective function $J(\theta)$. Two different optimization methods were used: (1) Stochastic Gradient Descent (SGD) and (2) Adaptive Moment Estimation (Adam).

Stochastic Gradient Descent SGD [16] allows optimizing model parameters by obtaining the loss for a mini-batch of images. Although this loss often substantially fluctuates from mini-batch to mini-batch, it can typically be minimized much faster thanks to a reduction in the number of calculations performed. Furthermore, the significant fluctuations in loss possibly also prevent SGD from getting stuck in a local minimum. The mathematical form of SGD using momentum[1] can be found below:

$$\begin{aligned} v_t &= \lambda \cdot v_{t-1} + g_t\,, \\ \theta_t &= \theta_{t-1} - \alpha \cdot v_t\,, \end{aligned} \tag{4}$$

where v_t represents the velocity, g_t represents the gradient, λ represents the momentum, and α represents the learning rate.

When making use of SGD, values for two hyperparameters need to be determined, namely for the momentum λ and the learning rate α. These hyperparameters were set to 0.9 and 0.001, respectively. The use of λ allows accelerating

[1] `https://pytorch.org/docs/stable/optim.html`

the optimization performed by SGD with less fluctuation. This is accomplished by multiplying the previous velocity with λ and adding the result to the current gradient g_t in order to obtain the current velocity v_t. The obtained current velocity is then used to update the parameters. The learning rate determines the step size made when updating the parameters in each iteration. It is important to set the learning rate adequately: an α-value that is too small leads to a slow convergence, whereas an α-value that is too large may cause divergence (overshoot).

Adaptive Moment Estimation Adam [17] is an adaptive learning rate optimizer, adopting the strengths of AdaGrad and RMSProp. When making use of Adam, the learning rate is computed individually for each parameter. Unlike SGD, which uses gradients directly, Adam utilizes exponentially moving averages of the gradient and the squared gradient. In particular, m_t is defined as the moving average of the gradient, which is an estimate of the first moment, whereas the current velocity of the Adam, u_t, is defined as the moving average of the squared gradient, which is an estimate of the second raw moment:

$$m_t = \beta_1 \cdot m_{t-1} + (1 - \beta_1) \cdot g_t \,, \tag{5}$$

$$u_t = \beta_2 \cdot u_{t-1} + (1 - \beta_2) \cdot g_t^2 \,. \tag{6}$$

In the above equations, β_1 and β_2 are the exponential decay rates for the first moment estimate and the second moment estimate, respectively (the default values $\beta_1 = 0.9$ and $\beta_2 = 0.999$ were used).

Both vectors of moving averages were initially set to 0. As training progresses, the contribution of the previous gradients to the moving average reduces, causing m_t and u_t to be biased towards zero. To avoid this bias, both m_t and u_t were corrected:

$$\widehat{m}_t = \frac{m_t}{1 - \beta_1^t} \,, \tag{7}$$

$$\widehat{u}_t = \frac{u_t}{1 - \beta_2^t} \,. \tag{8}$$

The parameters were then updated by re-scaling the learning rate using the bias-corrected estimators of the first and the second moment:

$$\theta_t = \theta_{t-1} - \frac{\alpha \cdot \widehat{m}_t}{(\sqrt{\widehat{u}_t} + \varepsilon)} \,. \tag{9}$$

The learning rate α was set to its default value of 0.001. Furthermore, ε, which allows avoidance of numerical issues, was set to 10^{-8}.

S2.5 Transfer learning

Deep neural network models consist of a large number of parameters that are trainable, and where training is achieved by using one or more datasets. The

trained parameters encode features that are meaningful for addressing a specific task such as image segmentation. The major drawbacks in training a deep neural network model are that it requires a significant amount of computational power and a substantial amount of data. Transfer learning [18] is a method for transferring the pre-trained parameters from a source model to a target model, where these parameters were previously used by the source model to perform a task that is similar to the task to be performed by the target model. In this context, the transferred parameters are typically fine-tuned with another dataset to learn features that are more relevant to the task to be addressed by the target model (in this study: MP segmentation from fluorescence microscopy images). By making use of transfer learning, the time needed to train a model can be reduced as the model does not have to be trained from scratch anymore.

In our study, transfer learning was utilized for constructing all TR-based deep learning models: the different U-Net variations and Nested U-Net were pre-trained with ImageNet [19], whereas FCN and DeepLabv3 were pre-trained with MS COCO 2017 [3].

References

[1] He K, Zhang X, Ren S, Sun J. Deep Residual Learning for Image Recognition. In: 2016 IEEE Conference on Computer Vision and Pattern Recognition (CVPR); 2016. p. 770–778.

[2] Russakovsky O, Deng J, Su H, Krause J, Satheesh S, Ma S, Huang Z, Karpathy A, Khosla A, Bernstein M, Berg AC, Fei-Fei L. ImageNet Large Scale Visual Recognition Challenge. International Journal of Computer Vision (IJCV). 2015;115(3):211–252. doi:10.1007/s11263-015-0816-y.

[3] Lin TY, Maire M, Belongie S, Hays J, Perona P, Ramanan D, Dollr P, Zitnick CL. Microsoft COCO: Common Objects in Context. In: Fleet D, Pajdla T, Schiele B, Tuytelaars T, editors. Computer Vision – ECCV 2014. Cham: Springer International Publishing; 2014. p. 740–755.

[4] Ronneberger O, Fischer P, Brox T. U-Net: Convolutional Networks for Biomedical Image Segmentation. In: Medical Image Computing and Computer-Assisted Intervention – MICCAI 2015. Springer International Publishing; 2015. p. 234–241.

[5] Minaee S, Boykov YY, Porikli F, Plaza AJ, Kehtarnavaz N, Terzopoulos D. Image Segmentation Using Deep Learning: A Survey. IEEE Transactions on Pattern Analysis and Machine Intelligence. 2021; p. 1–1. doi:10.1109/TPAMI.2021.3059968.

[6] Yakubovskiy P. Segmentation Models; 2019. `https://github.com/qubvel/segmentation_models`.

[7] Shelhamer E, Long J, Darrell T. Fully Convolutional Networks for Semantic Segmentation. IEEE Transactions on Pattern Analysis and Machine Intelligence. 2017;39(4):640–651. doi:10.1109/TPAMI.2016.2572683.

[8] Chen LC, Papandreou G, Kokkinos I, Murphy K, Yuille AL. DeepLab: Semantic Image Segmentation with Deep Convolutional Nets, Atrous Convolution, and Fully Connected CRFs. IEEE Transactions on Pattern Analysis and Machine Intelligence. 2018;40(4):834–848. doi:10.1109/TPAMI.2017.2699184.

[9] Chen LC, Zhu Y, Papandreou G, Schroff F, Adam H. Encoder-Decoder with Atrous Separable Convolution for Semantic Image Segmentation. Computer Vision – ECCV 2018 Lecture Notes in Computer Science. 2018; p. 833–851. doi:10.1007/978-3-030-01234-2_49.

[10] Zhou Z, Rahman Siddiquee MM, Tajbakhsh N, Liang J. UNet++: A Nested U-Net Architecture for Medical Image Segmentation. In: Stoyanov D, Taylor Z, Carneiro G, Syeda-Mahmood T, Martel A, Maier-Hein L, Tavares JMRS, Bradley A, Papa JP, Belagiannis V, Nascimento JC, Lu Z, Conjeti S, Moradi M, Greenspan H, Madabhushi A, editors. Deep Learning in Medical Image Analysis and Multimodal Learning for Clinical Decision Support. Cham: Springer International Publishing; 2018. p. 3–11.

[11] Huang G, Liu Z, Van Der Maaten L, Weinberger KQ. Densely Connected Convolutional Networks. In: 2017 IEEE Conference on Computer Vision and Pattern Recognition (CVPR); 2017. p. 2261–2269.

[12] Jadon S. A survey of loss functions for semantic segmentation. 2020 IEEE Conference on Computational Intelligence in Bioinformatics and Computational Biology (CIBCB). 2020;doi:10.1109/cibcb48159.2020.9277638.

[13] Sudre CH, Li W, Vercauteren T, Ourselin S, Cardoso MJ. Generalised Dice Overlap as a Deep Learning Loss Function for Highly Unbalanced Segmentations. In: Deep Learning in Medical Image Analysis and Multimodal Learning for Clinical Decision Support. Springer; 2017. p. 240–248.

[14] Milletari F, Navab N, Ahmadi SA. V-Net: Fully Convolutional Neural Networks for Volumetric Medical Image Segmentation. In: 2016 Fourth International Conference on 3D Vision (3DV. IEEE; 2016. p. 565–571.

[15] Deng R, Shen C, Liu S, Wang H, Liu X. Learning to Predict Crisp Boundaries. Computer Vision – ECCV 2018 Lecture Notes in Computer Science. 2018; p. 570–586. doi:10.1007/978-3-030-01231-1_35.

[16] Ruder S. An overview of gradient descent optimization algorithms. arXiv preprint arXiv:160904747. 2016;.

[17] Kingma DP, Ba J. Adam: A Method for Stochastic Optimization. arXiv preprint arXiv:14126980. 2014;.

[18] Tan C, Sun F, Kong T, Zhang W, Yang C, Liu C. A Survey on Deep Transfer Learning. In: International conference on artificial neural networks (ICANN). Springer International Publishing; 2018. p. 270–279.

[19] Krizhevsky A, Sutskever I, Hinton GE. ImageNet Classification with Deep Convolutional Neural Networks. Commun ACM. 2017;60(6):84–90. doi:10.1145/3065386.
