# Supplementary material for: MP-Net: Deep learning-based segmentation for fluorescence microscopy images of microplastics isolated from clams
Source: PLoS One. 2022 Jun 15;17(6):e0269449. doi: 10.1371/journal.pone.0269449 (PMC9200300; doi:10.1371/journal.pone.0269449)
Supplement: S3 File — (PDF) [file pone.0269449.s003.pdf]

S3. Learning curves

The learning curves of each model can be found in Figure 1 to Figure 9. For each model, a total of eight plots can be observed, four on the left and four on the right, corresponding to loss curves and performance curves, respectively. Since 4-fold cross-validation was performed, the curves for the four different fine-tuning steps are displayed one row at a time. The six variants of U-Net and Nested U-Net were fine-tuned for a total of 20 epochs. On the other hand, FCN and DeepLabv3 were fine-tuned for a total of only 10 epochs, given that the optimization time for these models was more than double that of the others.

As shown in Figure 2, the validation loss obtained for U-Net (2) is relatively flat, with the exception of the spike present in Fine-tuning (1). Similarly, U-Net (3) (Figure 3), U-Net (4) (Figure 4), TTA U-Net (3) (Figure 5), TTA U-Net (4) (Figure 6), and DeepLabv3 (Figure 8) exhibit a relatively stable validation loss. For FCN, there seems to be a slight decrease in validation loss, as shown in Figure 7. However, the performance scores obtained by FCN are generally lower than the ones obtained by the other models.

For U-Net (1) and Nested U-Net, the validation loss shows a generally increasing trend, as can be seen in the insets of Figure 1 and Figure 9, respectively. This increasing trend is particularly prominent for Nested U-Net. As more fine-tuning is applied, the recall of Nested U-Net noticeable decreases compared to the recall of the other models. Nevertheless, the best parameters were found for each fine-tuning step by taking into account the minimum validation loss. As a result, a high validation loss did not affect the final effectiveness of the models on the test set.

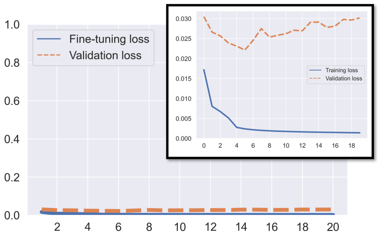

(a) U-Net (1): Fine-tuning (1) loss

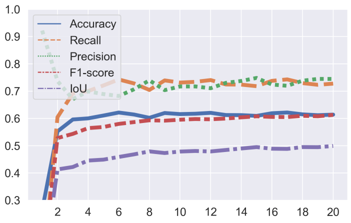

(b) U-Net (1): Fine-tuning (1) performance

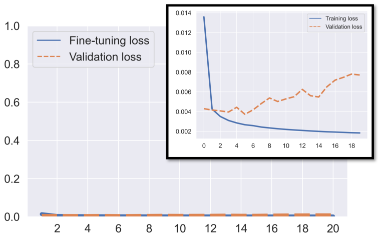

(c) U-Net (1): Fine-tuning (2) loss

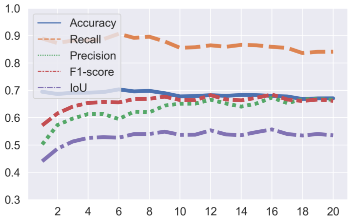

(d) U-Net (1): Fine-tuning (2) performance

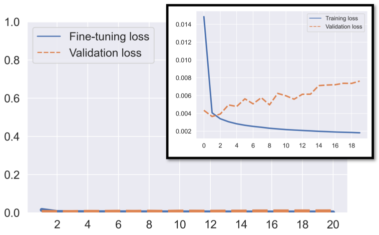

(e) U-Net (1): Fine-tuning (3) loss

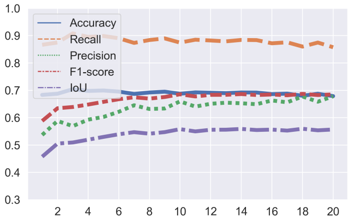

(f) U-Net (1): Fine-tuning (3) performance

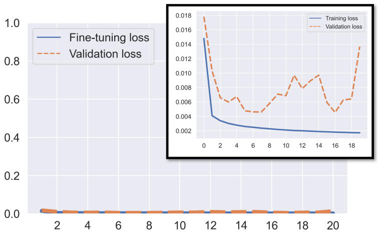

(g) U-Net (1): Fine-tuning (4) loss

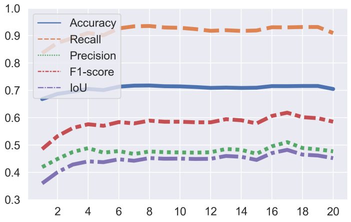

(h) U-Net (1): Fine-tuning (4) performance

Figure 1: U-Net (1) learning curves.

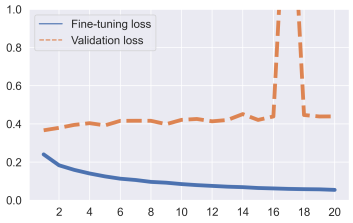

(a) U-Net (2): Fine-tuning (1) loss

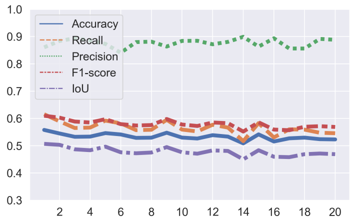

(b) U-Net (2): Fine-tuning (1) performance

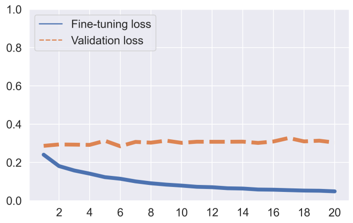

(c) U-Net (2): Fine-tuning (2) loss

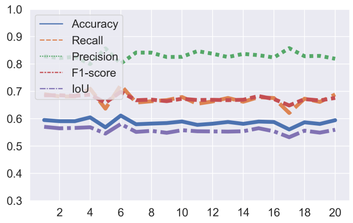

(d) U-Net (2): Fine-tuning (2) performance

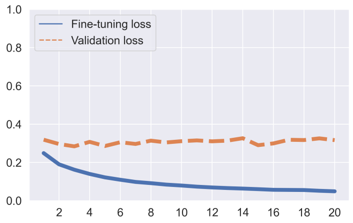

(e) U-Net (2): Fine-tuning (3) loss

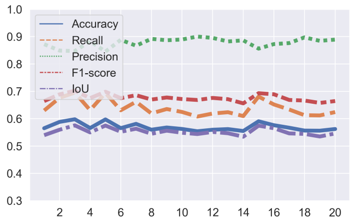

(f) U-Net (2): Fine-tuning (3) performance

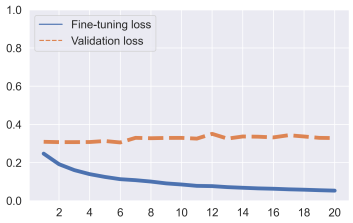

(g) U-Net (2): Fine-tuning (4) loss

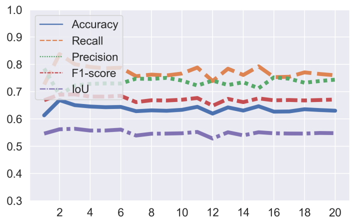

(h) U-Net (2): Fine-tuning (4) performance

Figure 2: U-Net (2) learning curves.

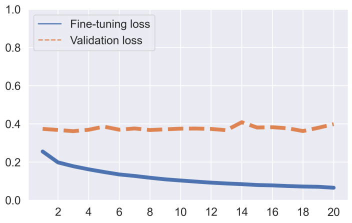

(a) U-Net (3): Fine-tuning (1) loss

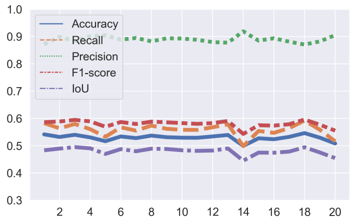

(b) U-Net (3): Fine-tuning (1) performance

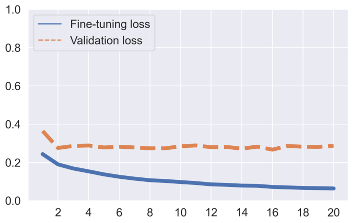

(c) U-Net (3): Fine-tuning (2) loss

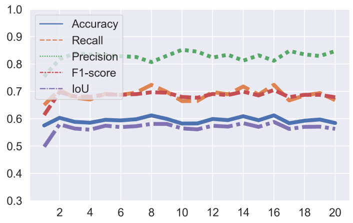

(d) U-Net (3): Fine-tuning (2) performance

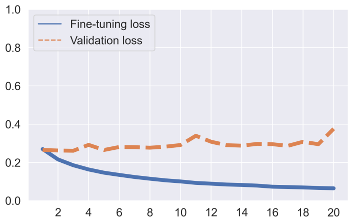

(e) U-Net (3): Fine-tuning (3) loss

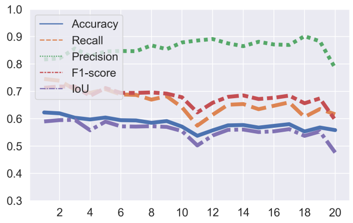

(f) U-Net (3): Fine-tuning (3) performance

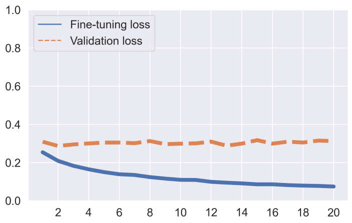

(g) U-Net (3): Fine-tuning (4) loss

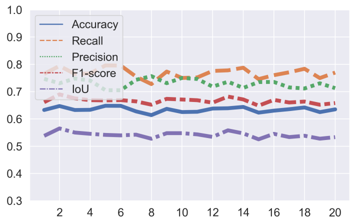

(h) U-Net (3): Fine-tuning (4) performance

Figure 3: U-Net (3) learning curves.

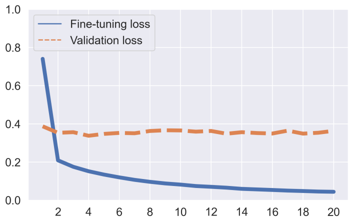

(a) U-Net (4): Fine-tuning (1) loss

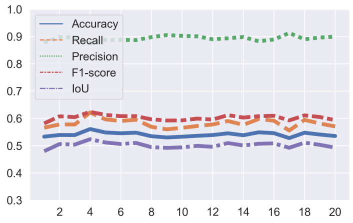

(b) U-Net (4): Fine-tuning (1) performance

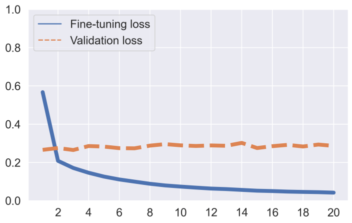

(c) U-Net (4): Fine-tuning (2) loss

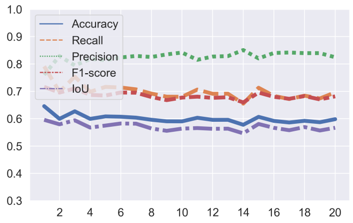

(d) U-Net (4): Fine-tuning (2) performance

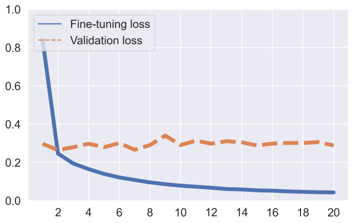

(e) U-Net (4): Fine-tuning (3) loss

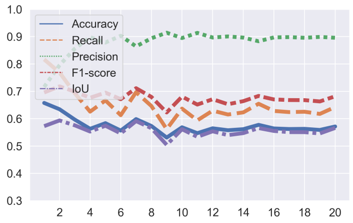

(f) U-Net (4): Fine-tuning (3) performance

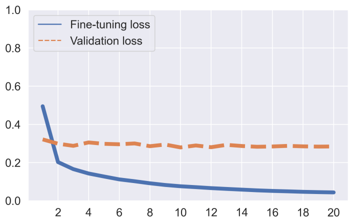

(g) U-Net (4): Fine-tuning (4) loss

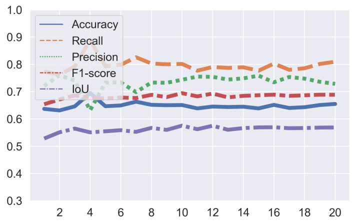

(h) U-Net (4): Fine-tuning (4) performance

Figure 4: U-Net (4) learning curves.

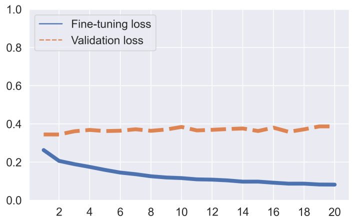

(a) TTA U-Net (3): Fine-tuning (1) loss

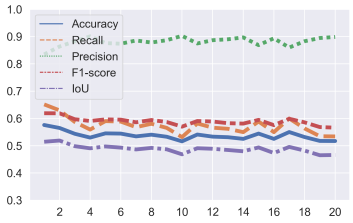

(b) TTA U-Net (3): Fine-tuning (1) performance

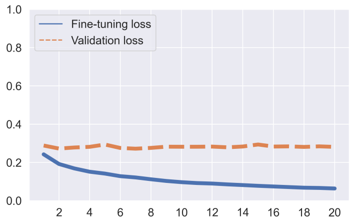

(c) TTA U-Net (3): Fine-tuning (2) loss

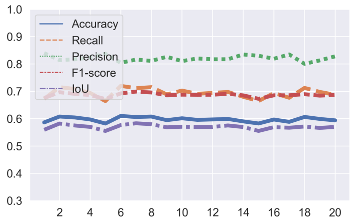

(d) TTA U-Net (3): Fine-tuning (2) performance

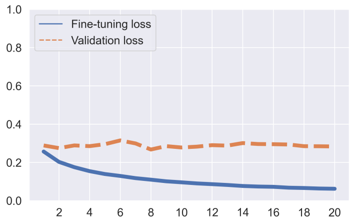

(e) TTA U-Net (3): Fine-tuning (3) loss

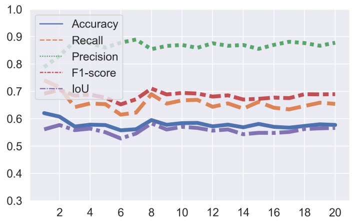

(f) TTA U-Net (3): Fine-tuning (3) performance

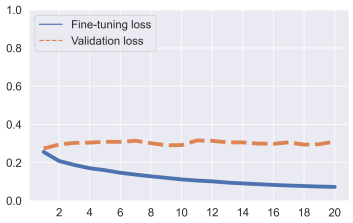

(g) TTA U-Net (3): Fine-tuning (4) loss

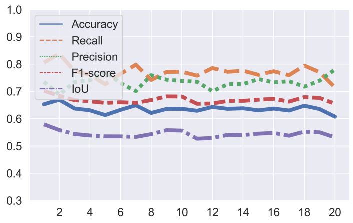

(h) TTA U-Net (3): Fine-tuning (4) performance

Figure 5: TTA U-Net (3) learning curves.

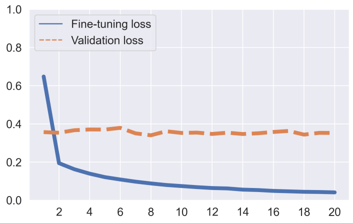

(a) TTA U-Net (4): Fine-tuning (1) loss

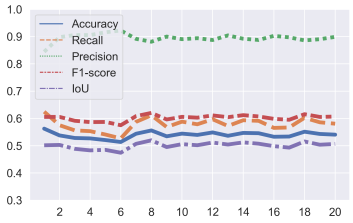

(b) TTA U-Net (4): Fine-tuning (1) performance

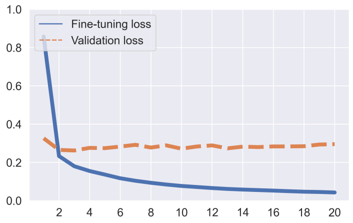

(c) TTA U-Net (4): Fine-tuning (2) loss

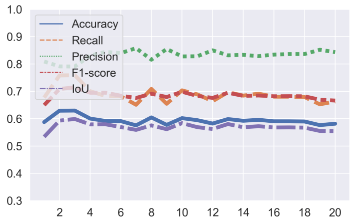

(d) TTA U-Net (4): Fine-tuning (2) performance

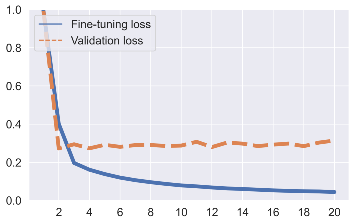

(e) TTA U-Net (4): Fine-tuning (3) loss

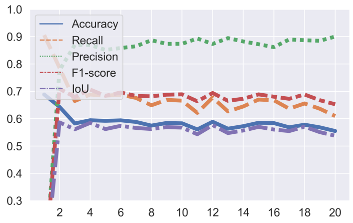

(f) TTA U-Net (4): Fine-tuning (3) performance

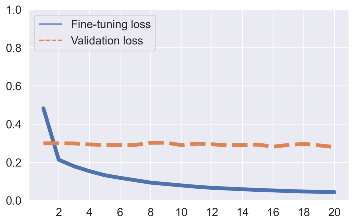

(g) TTA U-Net (4): Fine-tuning (4) loss

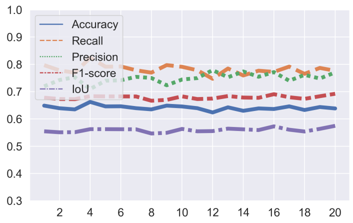

(h) TTA U-Net (4): Fine-tuning (4) performance

Figure 6: TTA U-Net (4) learning curves.

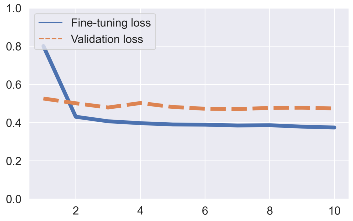

(a) FCN: Fine-tuning (1) loss

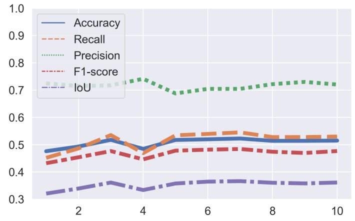

(b) FCN: Fine-tuning (1) performance

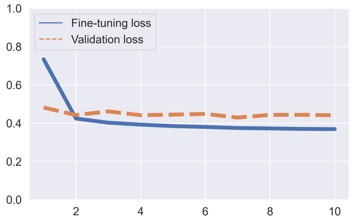

(c) FCN: Fine-tuning (2) loss

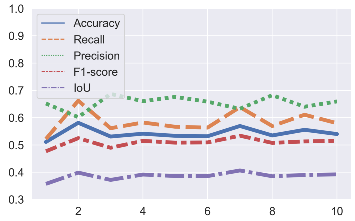

(d) FCN: Fine-tuning (2) performance

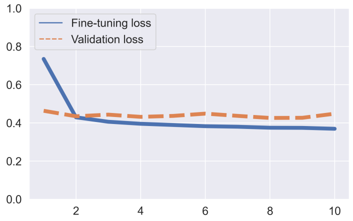

(e) FCN: Fine-tuning (3) loss

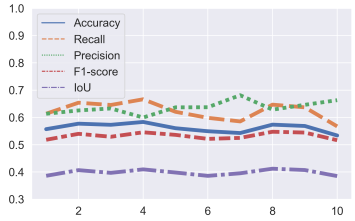

(f) FCN: Fine-tuning (3) performance

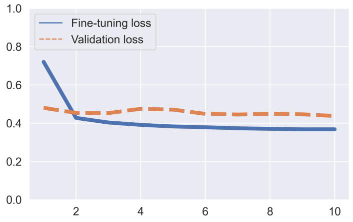

(g) FCN: Fine-tuning (4) loss

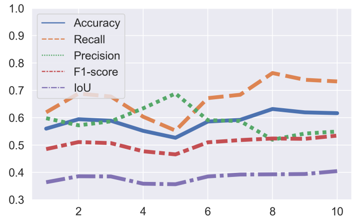

(h) FCN: Fine-tuning (4) performance

Figure 7: FCN learning curves.

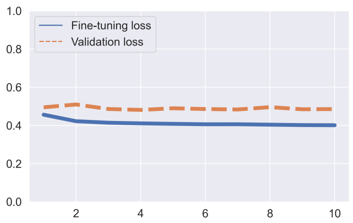

(a) DeepLabv3: Fine-tuning (1) loss

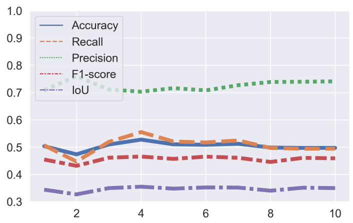

(b) DeepLabv3: Fine-tuning (1) performance

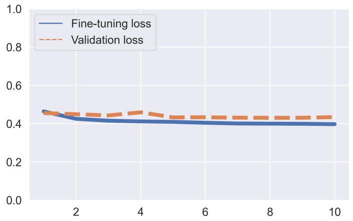

(c) DeepLabv3: Fine-tuning (2) loss

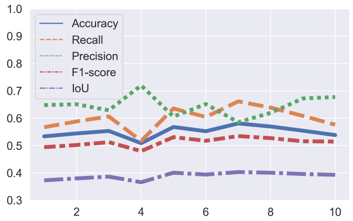

(d) DeepLabv3: Fine-tuning (2) performance

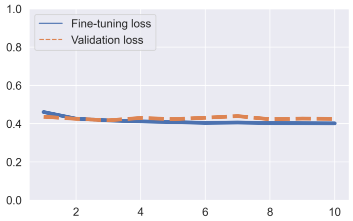

(e) DeepLabv3: Fine-tuning (3) loss

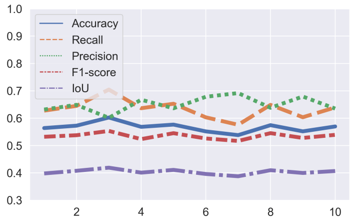

(f) DeepLabv3: Fine-tuning (3) performance

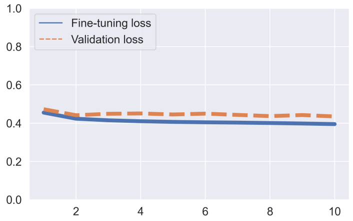

(g) DeepLabv3: Fine-tuning (4) loss

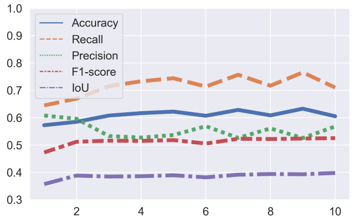

(h) DeepLabv3: Fine-tuning (4) performance

Figure 8: DeepLabv3 learning curves.

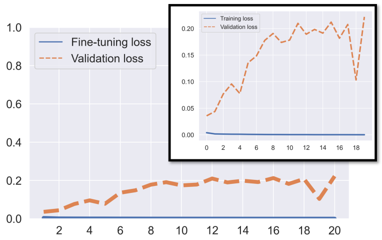

(a) Nested U-Net: Fine-tuning (1) loss

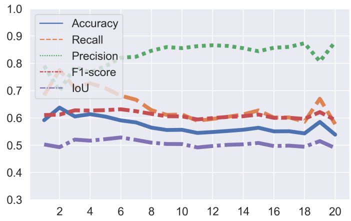

(b) Nested U-Net: Fine-tuning (1) performance

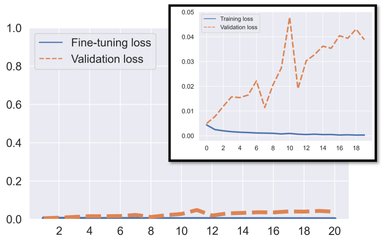

(c) Nested U-Net: Fine-tuning (2) loss

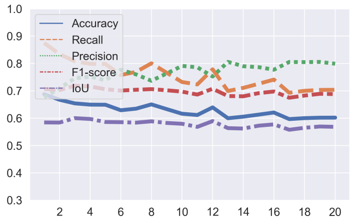

(d) Nested U-Net: Fine-tuning (2) performance

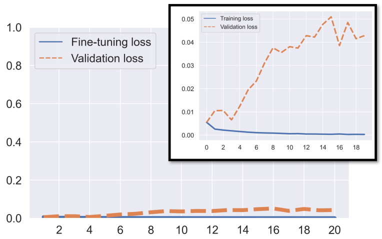

(e) Nested U-Net: Fine-tuning (3) loss

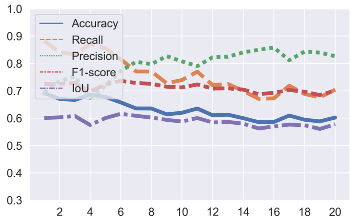

(f) Nested U-Net: Fine-tuning (3) performance

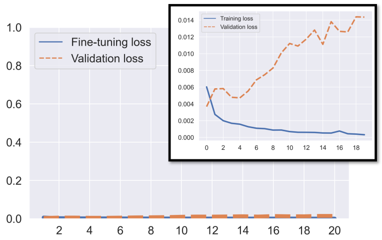

(g) Nested U-Net: Fine-tuning (4) loss

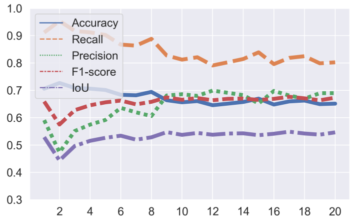

(h) Nested U-Net: Fine-tuning (4) performance

Figure 9: Nested U-Net learning curves.
