# Supplementary material for: MP-Net: Deep learning-based segmentation for fluorescence microscopy images of microplastics isolated from clams
Source: PLoS One. 2022 Jun 15;17(6):e0269449. doi: 10.1371/journal.pone.0269449 (PMC9200300; doi:10.1371/journal.pone.0269449)
Supplement: S4 File — (PDF) [file pone.0269449.s004.pdf]

S4. Testing phase

S4.1 Test-time augmentation

Augmentation [1], together with transfer learning [2], is a method that often helps deep learning in achieving high levels of effectiveness when having to work with a small-sized image dataset. Augmentation aims at making a model robust to different image changes by transforming images, used as an input for training, in various ways. Representative image augmentation methods include cropping, affine transformations, and color space changes.

After fine-tuning, augmentation can also be used in the testing phase. Specifically, several augmentations can be applied to the inputs used for testing, and the final prediction is then produced by combining the different predictions made. Through TTA, the chance of being over-confident towards incorrect predictions can be reduced. However, during testing, there is a drawback in terms of computational cost, given that the model under consideration needs to make predictions multiple times [1]. The use of TTA is known to have resulted in effectiveness improvements for VGG [3] and AlexNet [4], and it also helped in improving IoU values when performing segmentation [5]. In this study, we applied augmentation during both the fine-tuning and testing phase, denoting the resulting model as TTA U-Net. For model names without the prefix TTA, neither augmentation nor TTA was applied.

Figure 1 illustrates the image augmentation methods used in our experiments. When TTA was applied during fine-tuning, there was a 60% chance of using augmentation. Upon using augmentation, each of the five different augmentation methods was applied randomly. One the other hand, when using augmentation during testing, only three augmentation methods were applied to a particular patch, namely brightness, contrast, and HSV, thus resulting in three more patches. As a result, a total of four patches, including the original patch, were used to generate (i.e., predict) four corresponding mask patches. These mask patches were then averaged in a pixel-wise fashion to get the final mask patch.

S4.2 Model evaluation

To evaluate the effectiveness of our segmentation models, we used a total of five metrics: balanced accuracy, precision, recall, F_1-score, and IoU. Detailed descriptions of each metric can be found below.

Pixel accuracy is defined as the fraction of correct predictions (true positives and true negatives) over all predictions made:

$$\text{Pixel Accuracy} = \frac{\text{TP} + \text{TN}}{\text{TP} + \text{TN} + \text{FP} + \text{FN}} \,. \tag{1}$$

In the above equation, TP denotes the number of true positives, TN the number of true negatives, FP the number of false positives, and FN the number of false negatives.

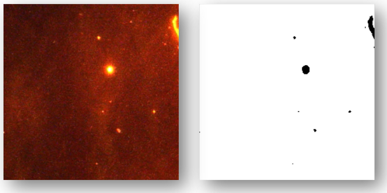

(a) Original

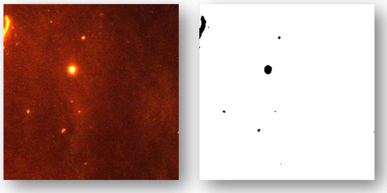

(b) Horizontal flip

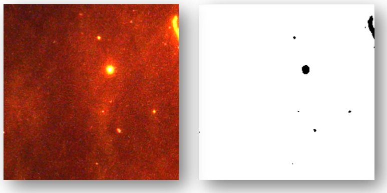

(c) Random contrast

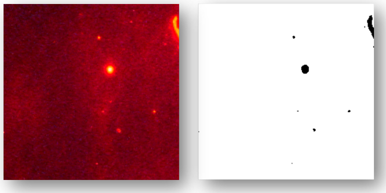

(d) Random hue saturation value (HSV)

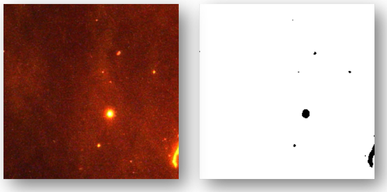

(e) Vertical flip

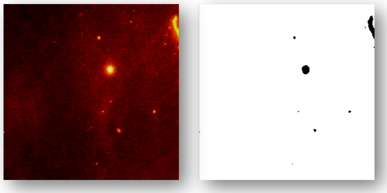

(f) Random brightness

Figure 1: Examples of image transformations used during (test-time) augmentation.

For imbalanced datasets, balanced accuracy is more suitable than pixel accuracy. The balanced accuracy is calculated as the average of the accuracy obtained for the positive class and the accuracy obtained for the negative class:

$$\text{Balanced Accuracy} = \frac{1}{2} \times \left(\frac{\text{TP}}{\text{TP} + \text{FN}} + \frac{\text{TN}}{\text{FP} + \text{TN}} \right) . \tag{2}$$

Since pixel accuracy includes the true negatives in the numerator, correctly classified background pixels lead to a high accuracy that is less sensitive to the number of true positives. Thus, precision and recall, which do not include the number of true negatives in their calculation, facilitate a more representative evaluation with regards to the number of correctly predicted MP pixels:

$$\begin{aligned} \text{Precision} &= \frac{\text{TP}}{\text{TP} + \text{FP}} , \\ \text{Recall} &= \frac{\text{TP}}{\text{TP} + \text{FN}} . \end{aligned} \tag{3}$$

The F_1-score, which is the harmonic mean of precision and recall, can also be used to evaluate the effectiveness of a segmentation model:

$$F_1\text{-score} = \frac{2 \times \text{Precision} \times \text{Recall}}{\text{Precision} + \text{Recall}} . \tag{4}$$

Finally, Intersection over Union (IoU), which is also known as the Jaccard Index, is defined as the area of intersection over the area of union between the ground truth and the predicted segmentation, with A and B referring to the ground truth and the predicted segmentation, respectively:

$$\text{IoU} = \frac{|A \cap B|}{|A \cup B|} = \frac{\text{TP}}{\text{TP} + \text{FN} + \text{FP}} . \tag{5}$$

Note that balanced accuracy, precision, recall, and F_1-score are calculated by taking the average of the mean performance over the different test sets from 4-fold cross-validation. In this case, the obtained balanced accuracy, recall, precision, F_1-score, and IoU are referred to as the mean balanced accuracy (mAccuracy), the mean recall (mRecall), the mean precision (mPrecision), the mean F_1-score (mF_1-score), and the mean IoU (mIoU), respectively.

References

[1] Shorten C, Khoshgoftaar TM. A survey on Image Data Augmentation for Deep Learning. Journal of Big Data. 2019;6(1). doi:10.1186/s40537-019-0197-0.

[2] Tan C, Sun F, Kong T, Zhang W, Yang C, Liu C. A Survey on Deep Transfer Learning. In: International conference on artificial neural networks (ICANN). Springer International Publishing; 2018. p. 270–279.

[3] Simonyan K, Zisserman A. Very Deep Convolutional Networks for Large-Scale Image Recognition; 2015. Available from: https://arxiv.org/abs/1409.1556.

[4] Krizhevsky A, Sutskever I, Hinton GE. ImageNet Classification with Deep Convolutional Neural Networks. Commun ACM. 2017;60(6):84–90. doi:10.1145/3065386.

[5] Saleh HM, Saad NH, Isa NAM. Overlapping Chromosome Segmentation using U-Net: Convolutional Networks with Test Time Augmentation. Procedia Computer Science. 2019;159:524–533. doi:10.1016/j.procs.2019.09.207.
