# Supplementary material for: MP-Net: Deep learning-based segmentation for fluorescence microscopy images of microplastics isolated from clams
Source: PLoS One. 2022 Jun 15;17(6):e0269449. doi: 10.1371/journal.pone.0269449 (PMC9200300; doi:10.1371/journal.pone.0269449)
Supplement: S5 File — (PDF) [file pone.0269449.s005.pdf]

S6. Additional results

S6.1 Impact of encoders

The TR-based deep learning models used in our study have an encoder for extracting features from the given input patches. In other words, an encoder can be seen as a core component of the aforementioned segmentation models. ResNet [1] is currently one of the most popular encoders to address computer vision-related tasks. All models used in our experiments are leveraging ResNet-101 as their encoder. For the experiment presented in this section, we selected MP-Net and we performed a comparative evaluation by introducing encoder changes. The results obtained are shown in Figure 1. Five different types of encoders were tested. The number next to each ResNet model refers to the number of stacked residual blocks. As discussed in [1], an increase in the number of stacked residual blocks leads,in general, to a higher effectiveness.

Among the encoders presented in Figure 1, ResNet-18 scored the highest in terms of mAccuracy, mRecall, and mIoU. On the other hand, ResNet-34 obtained the highest mPrecision and mF_1-score. Compared to ResNet-101, ResNet-34 consistently scored higher for all five metrics. Similarly, ResNet-18 outperforms ResNet-101 for all metrics, with the exception of mPrecision.

Our results suggest that MP-Net could be improved further by changing the encoder to ResNet-18 or ResNet-34. However, since the evaluation was performed using only CVF (1) as the validation set and CVF (2), CVF (3), and CVF (4) as the fine-tuning set (see Fine-tuning (1) in Figure 6, the execution of a complete cross-validation is desirable to confirm the obtained levels of effectiveness.

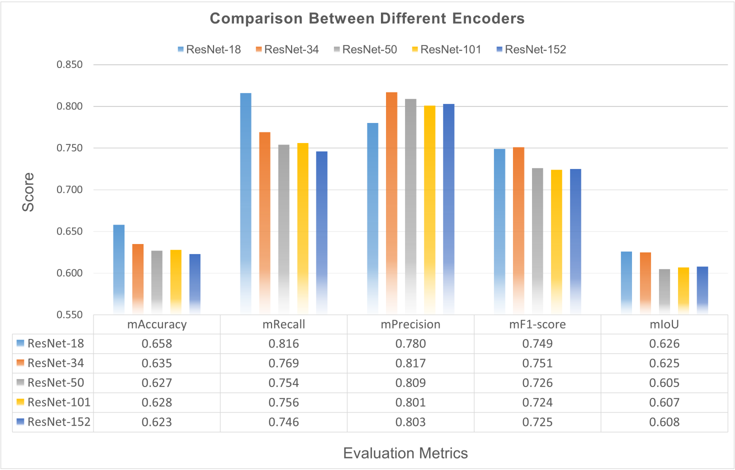

Figure 1: Effectiveness obtained by MP-Net when making use of different ResNet encoders. All models use Dice loss and the Adam optimizer. Note that the results are obtained based on Fine-tuning (1) only.

S6.2 Impact of test-time augmentation

This section describes the impact of TTA on the two most effective models: U-Net (3) and U-Net (4) (MP-Net).

The overall effectiveness obtained when making use of TTA can be found in Table 4. Also, the difference in effectiveness between the models, with and without TTA, is presented in Figure 2. A slight improvement in mPrecision of 3.25% can be observed for U-Net (3). The other metrics have decreased after application of TTA, with the largest decrease being 9.57% for mRecall. For U-Net (4), mAccuracy and mRecall increased by 1.20% and 2.11%, respectively. On the other hand, the effectiveness declined in terms of mPrecision, mF_1-score, and mIoU. Generally, there was a decrease in the effectiveness of both models using TTA; TTA seems to negatively affect the generalization ability of the models.

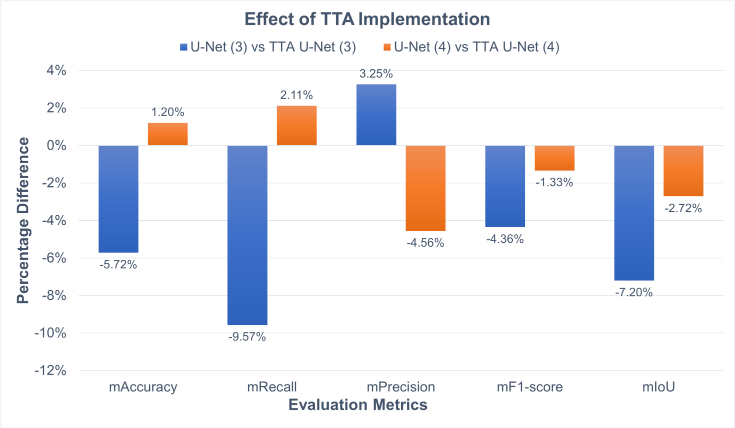

Figure 2: Comparison of the effectiveness, expressed as a difference in percentage, between U-Net (3) and U-Net (4), with and without implementation of TTA.

S6.3 Ablation study of image augmentation

As described in Supporting information S6.2, TTA has a negative impact on the effectiveness of the models it was added to. We attempted to find the reason behind this by conducting an ablation experiment. Random brightness (B), random contrast (C), and random hue saturation values (HSV), which affect the color distribution, were separately tested to determine which augmentation method adversely affected the final effectiveness. The best performing model, MP-Net, was selected for this study. The results obtained can be found in Figure 3.

When making use of single augmentations, B and C do not seem to greatly affect model effectiveness. However, the application of HSV resulted in reduced

mAccuracy, mRecall, mF_1-score, and mIoU values. When combining different types of augmentations, B+C scored the highest in terms of mAccuracy, mRecall, mF_1-score, and mIoU, even surpassing the combination B+C+HSV. Since these results are the outcome of an evaluation that was limited in scope, specifically, using only Fine-tuning (1) (see Figure 6), performing complete cross-validation is necessary to confirm the effects of combining different image augmentation methods. Nevertheless, the use of augmentations opened up the possibility of further improving model effectiveness.

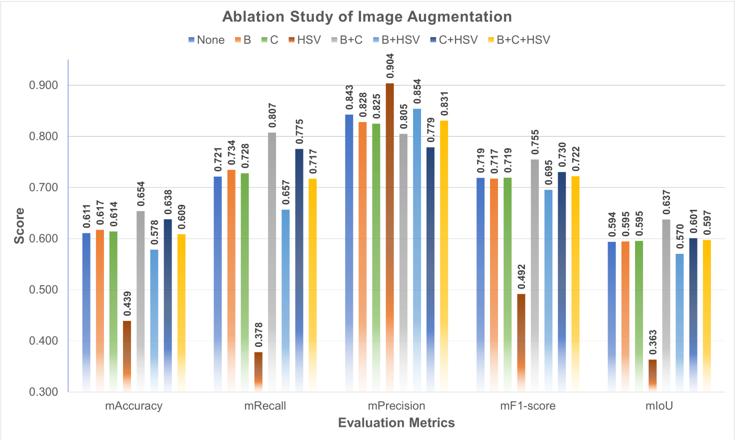

Figure 3: Ablation study of image augmentation for MP-Net. None: no image augmentation, B: random brightness, C: random contrast, HSV: random hue saturation values. Note that the results were obtained based on only Fine-tuning (1).

References

[1] He K, Zhang X, Ren S, Sun J. Deep Residual Learning for Image Recognition. In: 2016 IEEE Conference on Computer Vision and Pattern Recognition (CVPR); 2016. p. 770–778.
